# The Domino Effects of Federal Research Funding

**DOI:** 10.1371/journal.pone.0157325

**Published:** 2016-06-21

**Authors:** Lauren Lanahan, Alexandra Graddy-Reed, Maryann P. Feldman

**Affiliations:** 1 Department of Management, Lundquist College of Business, University of Oregon, Eugene, Oregon, United States of America; 2 Price School of Public Policy, University of Southern California, Los Angeles, California, United States of America; 3 Department of Public Policy, University of North Carolina at Chapel Hill, Chapel Hill, North Carolina, United States of America; 4 Science of Science Innovation and Policy, Directorate of Social, Behavioral & Economic Sciences, National Science Foundation, Arlington, Virginia, United States of America; Iowa State University, UNITED STATES

## Abstract

The extent to which federal investment in research crowds out or decreases incentives for investment from other funding sources remains an open question. Scholarship on research funding has focused on the relationship between federal and industry or, more comprehensively, non-federal funding without disentangling the other sources of research support that include nonprofit organizations and state and local governments. This paper extends our understanding of academic research support by considering the relationships between federal and non-federal funding sources provided by the National Science Foundation Higher Education Research and Development Survey. We examine whether federal research investment serves as a complement or substitute for state and local government, nonprofit, and industry research investment using the population of research-active academic science fields at U.S. doctoral granting institutions. We use a system of two equations that instruments with prior levels of both federal and non-federal funding sources and accounts for time-invariant academic institution-field effects through first differencing. We estimate that a 1% increase in federal research funding is associated with a 0.411% increase in nonprofit research funding, a 0.217% increase in state and local research funding, and a 0.468% increase in industry research funding, respectively. Results indicate that federal funding plays a fundamental role in inducing complementary investments from other funding sources, with impacts varying across academic division, research capacity, and institutional control.

## Introduction

In 2014, $52.2 billion was invested in academic science research at U.S. doctoral granting institutions. This funding was provided by multiple sources to further specific objectives. While industry aims to create new products and innovations that will spur commercial benefit, state and local governments invest to realize tangible economic benefits within their borders, and nonprofit organizations invest to create public benefits that will improve societal welfare. The federal government, however, accounts for the largest share of investment with the broader objectives of promoting mission agency mandates and conducting research important for national economic priorities.

The presence of multiple stakeholders prompts a debate over the extent to which different funding sources are complements or substitutes. Members of the U.S. Congress debate the extent to which federal investment in research crowds out or decreases incentives for investment from other funding sources. Under this scenario, federal funding yields no net increase in R&D investment and therefore is not a good use of taxpayer funds. The alternative view is that federal investment supports high-risk research that typically has long time horizons and induces additional investment by other funding sources. According to this view, federal investment crowds in complementary funding from non-federal sources.

Studies examining this phenomenon highlight the methodological concerns over sample selection bias and causal identification [1]. Selection bias, in particular, accounts for the lack of consensus on the effect of government spending on firm level R&D investment activity [1–2] due to limited access to detailed R&D financing data for proprietary firms. Another limitation has been the prominent focus on the relationship between federal and industry or federal and total non-federal sources of R&D funding [3–4]. Prior to 2010, the National Science Foundation’s (NSF) Survey of Research and Development Expenditures at Universities and Colleges provided expenditure data by five categories: federal, state and local government, industry, university-own institution, and the catch all *other* category. However, in 2010 the survey was redesigned as the NSF Higher Education Research and Development (HERD) Survey. Currently, five annual panels of institution-field level R&D data are available (2010–2014), which provide an unprecedented ability to examine the fuller range of research funders at the more granular level of the academic field within the institution. Moreover, nonprofit funders are now a distinct category.

Taken together, this paper aims to address these past limitations and contribute to the scholarship with the following:

1. Relying on the recent and detailed NSF HERD survey data, we are able both to understand the more detailed role of a fuller range of non-federal funders and to examine their relationships.
2. We are able to avoid sample selection bias by examining the population of academic science fields with active federal funding at U.S. doctoral-granting institutions.
3. By analyzing academic fields–arguably analogous to academic departments–we provide a more detailed understanding of funding relationships at the unit of research production.
4. Employing an instrumental variables estimation procedure, we can more appropriately isolate the relationship between funding sources. We use a dynamic panel model to address identification by instrumenting with prior levels of both federal and non-federal funding sources.

This approach produces empirical evidence to advance our understanding of the relationship between research funders investing in science fields at academic institutions. We find that federal funding plays a fundamental role in inducing complementary investments from other funding sources. Our analysis reveals that impacts vary across broad divisions of academic science fields, public versus private institutional control, and academic field research capacity. The evidence suggests that federal dollars are crowding in investments from other non-federal funders to achieve broad societal objectives.

## Data and Research Design

The NSF HERD Survey asks university respondents to apportion annual research expenditures to academic fields by research funding source. The fields loosely mirror academic departments. The funding sources include the federal government, state and local governments, nonprofit organizations, university-own funds, industry, and other sources. The latter category of *other* includes foreign support and individual sponsorship. While universities self-report the funding allocations, we draw upon this dataset given that it serves as the primary source for the portfolio of research expenditures within U.S. higher education institutions (http://www.nsf.gov/statistics/srvyherd/#qs&sd).

We analyze 26 standard science fields, which we assign to six broad academic divisions–Life Sciences, Engineering, Mathematical and Computer Sciences, Physical Sciences, Environmental Sciences, and Social Sciences and Psychology. Table 1 shows the crosswalk between the academic divisions and fields. For the analysis, we draw the sample from U.S. doctoral-granting academic institutions as defined by the National Center for Science and Engineering Statistics (NCSES) based on the classification of “doctoral-granting” institutions from the variable “highest degree granted.” We exclude specialized institutions with only a medical or engineering focus as identified by the NSF’s Web Computer-Aided Science Policy Analysis and Research (WebCASPAR) database.

**Table 1:** Crosswalk between Academic Division and Academic Field.

Academic Division	Academic Field
Engineering	Aerospace Engineering, Chemical Engineering, Civil Engineering, Electrical Engineering, Materials Engineering, Mechanical Engineering, Other Engineering
Environmental Sciences	Atmospheric Sciences, Earth Sciences, Oceanography, Other Geosciences
Life Sciences	Agricultural Sciences, Biological Sciences, Medical Sciences, Other Life Sciences
Mathematical and Computer Sciences	Mathematics and Statistics, Computer Science
Physical Sciences	Astronomy, Chemistry, Other Physical Sciences, Physics
Social Sciences and Psychology	Psychology, Economics, Political Science and Public Administration, Sociology, Other Social Sciences

*Notes*: The crosswalk follows NSF’s S&E Field Classification. For this analysis we group together Mathematics with Computer Sciences and Psychology with Social Sciences. *Source*: http://www.nsf.gov/statistics/rdexpenditures/glossary/s_efield.htm

**Fig 1:**
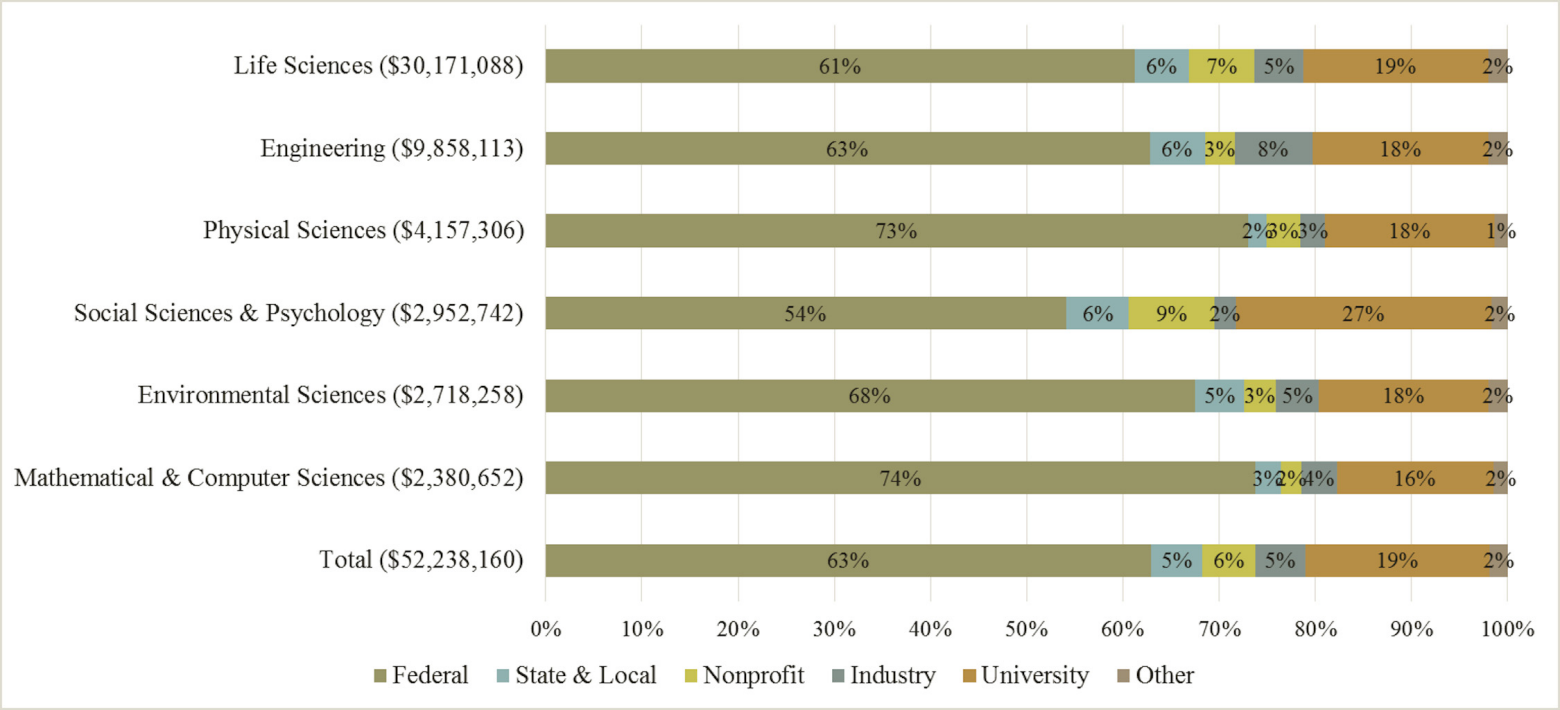
Distribution of R&D Funding Source by Broad Field, 2010–2014. *Notes*: Percentages reflect the distribution of funding sources based on average division funding levels (adjusted for inflation) from 2010 to 2014. Dollar values listed in parentheses are the nominal total university funding in 2014 ($1,000s) by division.

There is great diversity among science fields both in terms of the amount of money received and the share from alternative funding sources. Fig 1 presents stacked bar charts for the distribution of research expenditures by the sources tracked by the NSF HERD Survey for the six broad academic divisions. The distribution is based on the average funding from 2010 to 2014. Overall, federal funding accounts for 63% of academic R&D, while industry, state and local, and nonprofits each contribute between 5% and 6%. This distribution, however, varies across the six broad science divisions. For example, federal funding provides almost three-quarters of funding in the physical sciences (73%) and mathematics and computer sciences (74%). However, the proportion of federal funding is 54% for the social sciences and psychology. Regarding non-federal sources, nonprofits account for 9% of funding in the social sciences and psychology, while industry funds 8% of research for engineering.

University-own (internal) funds account for 19% of research overall, with a range of 16% for mathematical and computer sciences to 27% for social sciences and psychology. University-own funding is difficult to categorize because it is a combination of interest income from endowment, gifts, bequests, and other contributions to the university that are not counted as sponsored research but are subsequently allocated to research funding. These funds are often used as start-up packages to faculty or internal competitions. Use of university-own funds is higher for public universities (22%) than in private universities (12%). Interviews with university sponsored research offices and development offices revealed variation in what is reported in this category, suggesting that this category is itself somewhat of a catchall that warrants additional investigation that is beyond this paper. For this analysis, we exclude this source of funding and estimate the relationship between five funding sources: federal government, state and local government, nonprofit organizations, industry, and other.

With an explicit focus of examining the effect of federal funding on a range of other non-federal sources, we only include observations for academic fields with an active *federal* funding stream over the entire five-year panel (2010–2014) of the NSF HERD survey. Observations are at the institution-field level, and offer a more granular unit of analysis at the level of research production–arguably analogous to academic departments. Given this focus, the sample is drawn from the more research-active doctoral granting U.S. institutions. This sample of active, federally-funded institution-fields accounts for 35 percent of the population of fields and roughly 91 percent of federal research funding reported in the five-year panel of the NSF HERD survey. Additional research might examine factors that account for funding variation among the entire population of science fields; however, we narrow the sample given the focus of our primary research question.

While each academic field in our sample reports a continuous stream of active federal funding, a portion of these fields lacks consistent funding from the non-federal sources. The percentage of zero observations in the panel is 40% for state and local government, 30% for nonprofit organizations, and 33% for industry funding. Given that parameters for sample selection are determined by federal funding activity, we include these observations in the analysis. Moreover, we adjust all funding levels to account for inflation using the Gross Domestic Product Implicit Price Deflator, with 2009 as the base year. We then use the natural log form in *all* estimations. The NSF HERD Survey reports funding data in $1,000s, which we adjust before taking the natural log. For observations with zero non-federal funding values, we set the level to one before computing the natural log to ensure the natural log is equivalent to zero.

The sample consists of 3,460 unique institution-field observations (indexed by institution *n* and field *i*) from 266 institutions. The median number of science fields represented per institution is 13 (out of a possible 26, refer to Table 1 for full list) with a standard deviation of 6.5. With a five-year, balanced panel, this yields a total of 17,300 institution-field-year observations. The supplementary information–S1 File–annotates the entire data building process and empirical techniques presented in the paper. The code for both components–data building and empirical analysis–are publicly available online. In addition, we have uploaded the cleaned dataset. This information is available at: doi:10.7264/N3W957G6 <http://hdl.handle.net/1794/19409>.

### Estimation Method

We are interested in estimating the effect of federal R&D funding on a series of non-federal sources. Formally, we express this relationship with the function:

$$Y_{int}=f\left(X_{int},Y_{int-1},Z_{int},A_{t},\alpha_{in}\right)$$

where *i* denotes the academic field, *n* indexes the institution, and *t* is the annual time period. *Y* is the outcome variable of the non-federal funding source of interest. We estimate models for three outcomes: state and local, nonprofit, and industry R&D funding. *X* delimits the key explanatory variable–federal R&D funding. *Z* denotes the set of non-federal funding that excludes *Y*–the outcome variable being estimated. This controls for fluctuations in the broader R&D funding portfolio that may cause spurious correlation with the primary relationship of interest. *A* captures annual general macroeconomic shocks that might affect R&D funding streams. *α* is an institution-field fixed effect to account for time-invariant factors, which is essential as academic settings constitute highly institutionalized, organizational fields that are resistant to change [5–6]. Lastly, we include the one-year lagged dependent variable, *Y*_*t*−1_, to control for prior capacity to secure the non-federal funding outcome.

We are interested in the relationships between these different funding sources, which are however, endogenous and jointly determined. Inclusion of the one-year lagged dependent variable and fixed effects estimators alone, though, does not obviate endogeneity as the lagged component, *Y*_*int*−1_, is correlated with the error component, *ε*_*int*−1_, in the fixed effects model [7]. In their seminal paper, Arellano and Bond [8] offer a resolution by instrumenting the lagged dependent variable at least two periods in the fixed effects model. This work has served as the foundation for a larger body of scholarship on dynamic panel models [9–12]. As an extension, Blundell and Bond [13] advanced this method by developing an additional approach to increase the efficiency of the model by instrumenting levels with first differences rather than relying on standard fixed effects [11].

We draw upon these methods to include both first differences and the instrumented lagged dependent variable. In addition, dynamic panel models also utilize a set of instruments to account for endogeneity of prior trends of independent variables. Given that federal R&D funding has historically high and relatively stable levels of research investment [14], we treat this regressor as predetermined, which assumes that it is correlated with past errors, but uncorrelated with future errors. Federal funding is then instrumented with the following lags: *X*_*int*−1_, …, *X*_*int*−4_. [11, 13].

We also instrument for the additional contemporaneous non-federal funding activity, which we expect to be influenced by federal funding levels and potentially to influence each other. If excluded, this could confound the primary relationship of interest. For example, changes in industry-funded research may influence federal funding investment for the field of engineering, causing a spurious correlation between nonprofit and federal funding if industry is omitted. Table 2 presents the set of controls, *Z*, for each model.

**Table 2:** R&D Outcome Source and Respective Controls.

R&D Outcome Source (Y)	Controls of Non-Federal Funding Sources (Z)
Nonprofit	State & Local, Industry, Other
State & Local	Nonprofit, Industry, Other
Industry	Nonprofit, State & Local, Other

Given that we assume each of our outcomes of non-federal funding are endogenous, we also estimate the vector of additional non-federal regressors with multiple lags starting with the two year lag, *Z*_*int*−2_, …, *Z*_*int*−4_, for each source indicated in Table 2 [15].

Taken together, this instrumental variables approach conditions on the following: (i) first differences to control for institution-field specific variation; (ii) the lagged dependent regressor to address endogeneity of the non-federal funding outcome; and (iii) other, contemporaneous non-federal funding activity that cause spurious correlation. While other studies have relied on this model [16–17], to our knowledge this method has not been applied in this line of scholarship on R&D funding relationships spanning the public and private spheres. The supplementary notation–S2 File–presents a more detailed explanation and additional motivation of the primary estimation method.

Eq 1 presents the primary dynamic panel model, where *i* denotes the field, *n* denotes the institution, and *t* denotes the year. Eqs 1.1–1.3 expand the estimations for each set of instruments. *w* denotes the instrument for Eqs 1.1–1.3. All funding sources are estimated in log form.

$$\Delta Y_{int}=\beta_{1}(\Delta X_{int})+\beta_{2}\left(\Delta Y_{int-1}\right)+\beta_{z}\left(\Delta Z_{int}\right)+A_{t}+\Delta \varepsilon_{int}$$ (1)

where,

$$\Delta X_{int}=\delta_{1}+\sum_{l=1}^{4}(\delta_{w1,l}\left(X_{int-l}\right))+\varepsilon_{2int}$$ (1.1)

$$\Delta Y_{int-1}=\delta_{2}+\sum_{k=2}^{4}(\delta_{w2,k}\left(Y_{int-k}\right))+\varepsilon_{3int}$$ (1.2)

$$\Delta Z_{int}=\delta_{3}+\sum_{k=2}^{4}(\delta_{w3,k}\left(Z_{int-k}\right))+\varepsilon_{4int}$$ (1.3)

and where l ranges from 1 to 4 (l≥1) and *k* ranges from 2 to 4 (*k* ≥ 2), thus each regressor is instrumented with multiple lags. The Model Specification I in the supplementary materials–S3 File–presents the complete notation for these three sets of equations, respectively. Given that we estimate contemporaneous R&D funding activity, we do not claim to estimate causality with this model. However, this approach offers advantages by controlling for factors that confound the results.

## Results and Discussion

Table 3 presents results for the instrumental variables model (Eq 1) for the full sample for each outcome. Column 1 presents the results for the relationships with state and local government funding, while Column 2 presents the results for the relationships with nonprofit organizations and Column 3 for industry. The standard errors are clustered by institution-field to account for autocorrelation; this is the most granular unit available within this dataset. All funding sources are estimated in log form. In estimating the marginal effect for these log-log models, the coefficients are reported as the elasticity or responsiveness of non-federal sources to a change in federal or other funding levels. Note that elasticity is interpreted as a 1% change in the X variable being associated with a Beta% change in the Y variable. For these estimations, a 1% increase in federal funding is associated with a Beta% change in the funding level of state and local, nonprofit, or industry.

**Table 3:** Dynamic Panel Model Results by Outcome (Eq 1).

Variables	(1)	(2)	(3)
	State & Local	Nonprofit	Industry
*Logged Funding Source*			
Federal	0.217***	0.411***	0.468***
	(0.077)	(0.073)	(0.076)
State & Local		0.045***	0.006
		(0.014)	(0.030)
Nonprofit	0.036**		0.031**
	(0.015)		(0.015)
Industry	0.075**	0.139***	
	(0.036)	(0.036)	
Other	0.001	0.033**	0.069***
	(0.013)	(0.013)	(0.013)
One Year Lagged Logged Dependent Variable	0.527***	0.460***	0.428***
	(0.020)	(0.018)	(0.019)
Observations	13,840	13,840	13,840
Number of Institution-fields	3,460	3,460	3,460
Year Dummies Included	Yes	Yes	Yes
First Differences	Yes	Yes	Yes
*Post-specification Test Results*			
Serial Correlation AR(2) Pr > z	Fail to Pass	Pass	Pass
Sargan/Over-Identification (Prob > chi2)	Fail to Pass	Fail to Pass	Fail to Pass

*Notes*: Column 1 presents results with state and local R&D as the outcome (referring to Equations A and A.1 – A.5 in supplementary materials–S3 File); Column 2 presents results with nonprofit R&D as the outcome (referring to Equations B and B.1 – B.5 in supplementary materials–S3 File); Column 3 presents results with industry R&D as the outcome (referring to Equations C and C.1 – C.5 in supplementary materials–S3 File); clustered standard errors in parentheses (clustered by institution-field)

*** p<0.01

** p<0.05

* p<0.1. All funding is adjusted to FY 2009 and estimated in log form; dependent variable is logged funding of specified type; coefficients are elasticities. Federal funding is treated as predetermined and uses time t – l, where l≥1, to instrument for t; the set of other funding sources are treated as endogenous and use time t–k, where k ≥ 2, to instrument for t; the lagged dependent variable is treated as endogenous and uses time t–k, where k ≥ 2, to instrument for t– 1.

The coefficient for federal funding activity–the primary explanatory variable–is positive and statistically significant across each outcome, providing consistent evidence of a complementary relationship. The largest complementary relationship is for industry such that a 1% increase in federal R&D funding is associated with a 0.468% increase in the amount of industry R&D funding, on average. A 1% increase in federal R&D funding is also associated with a 0.411% increase in funding by nonprofits and a 0.217% increase in funding by state and local governments.

Table 3 also provides evidence of complementarity between the sources of non-federal R&D funding and the respective outcome. Across all three outcomes, the set of additional non-federal funding sources exhibits a positive and statistically significant relationship with the exception of other funding on state and local (Col. 1) and of state and local on industry funding (Col. 3). However, the size of the effect for the primary explanatory variable is an order of magnitude larger than the additional contemporaneous sources of non-federal research.

There are two post specification tests available to determine if the error terms across years are serially uncorrelated and if the lagged instruments meet the test of over-identifying restrictions, respectively [11]. For the former, we estimate whether Δ*ε*_*int*_ are correlated with Δ*ε*_*int*−*k*_ for k = 2. While this test measures for k ≥ 2, we are restricted, since we only have a five year panel. This is calculated based on the correlation of fitted residuals $\Delta \hat{\varepsilon_{int}}$. For the latter, we rely on the Sargan statistic to estimate if the population moment conditions are correct [15] (pg. 301). As noted in Table 3, the estimation satisfies the first test of serial correlation for the outcomes of nonprofit and industry funding, but not for state and local funding. The results fail to pass the Sargan test of over-identification for each outcome. This statistic, however, is prone to weakness with dynamic panel models given that it grows weaker as the number of instruments increases [11, 18]. For each set of estimations presented above the number of instruments is 61. Moreover, as Sargan [19] noted in his seminal work, the validity of the test is “proportional to the number of instrumental variables, so that, if the asymptotic approximations are to be used, this number must be small” (pg. 393). We recognize that this is a tradeoff for using a model reliant on multiple instruments.

### Additional Model Specifications

Because of the policy importance of the results, we estimate a series of additional model specifications to assess consistency of the results estimated by the primary model (Eq 1). These include the following: (i) academic institution-field and year fixed effects model (Eq 2), (ii) pooled, cross-sectional OLS model with the inclusion of two lagged logged dependent variables as regressors: *Y*_*int*−1_ and *Y*_*int*−2_ (Eq 3) where the standard errors are clustered at the institution-field level; and (iii) an alternate dynamic panel model that defines the vector of regressors, *Z*_*int*_, as *predetermined* rather than endogenous (Eq 4). For the set of equations listed below, *i* denotes the field, *n* denotes the institution, and *t* denotes the year.

$$Y_{int}=\alpha_{in}+\;\beta_{1}(X_{int})+\beta_{w}(Z_{int})+A_{t}+\varepsilon_{int}$$ (2)

$$Y_{int}=\beta_{0}+\beta_{1}(X_{int})+\beta_{2}(\;Y_{int-1})+\beta_{3}(\;Y_{int-2})+\beta_{x}\left(Z_{int}\right)+A_{t}+\varepsilon_{int}$$ (3)

Regarding Eq 2 and Eq 3, Angrist and Pischke [7] highlight that the conditions for consistent estimation of the lagged instrument in the instrumental variables model (Eq 1) are more demanding having more stringent assumptions than the fixed effects model or lagged dependent variable model alone (p. 245). Nevertheless, we present the results from Eq 2 and Eq 3 given that they estimate fundamental components of the primary model (Eq 1) and offer useful benchmarks.

As a third additional model specification, we relax the assumptions for the instruments in the dynamic panel model. While our primary model (Eq 1) estimates the elasticities by treating the set of regressors *Z*_*int*_ as endogenous (Eq 1.3), we relax that assumption and instead treat these measures as predetermined where the vector of regressors is each instrumented by $Z_{it-l}$ rather than *Z*_*it*−*k*_ (where l ranges from 1 to 4 (l≥1) and *k* ranges from 2 to 4 (*k* ≥ 2)). With this approach, the number of instruments increases (from 61 to 65). Eq 4 presents the generalized notation and includes the estimations for the endogenous (Eq 4.2) and predetermined (Eq 4.1 and Eq 4.3) instrumental variables. *w* denotes the instrument for Eq 4.1–4.3.

$$\Delta Y_{int}=\beta_{1}(\Delta X_{int})+\beta_{2}\left(\Delta Y_{int-1}\right)+\beta_{z}\left(\Delta Z_{int}\right)+A_{t}+\Delta \varepsilon_{int}$$ (4)

where,

$$\Delta X_{int}=\delta_{1}+\sum_{l=1}^{4}(\;\delta_{w1,l}\left(X_{int-l}\right))+\varepsilon_{2int}$$ (4.1)

$$\Delta Y_{int-1}=\delta_{2}+\sum_{k=2}^{4}(\;\delta_{w2,k}\left(Y_{int-k}\right))+\varepsilon_{3int}$$ (4.2)

$$\Delta Z_{int}=\delta_{3}+\sum_{l=1}^{4}(\;\delta_{w3,l}\left(Z_{int-l}\right))+\varepsilon_{4int}$$ (4.3)

As with the primary model, the supplementary materials–S3 File–presents each complete set of equations in the Model Specification sections II, III, and IV for Eqs 2, 3 and 4, respectively. Again, to be clear, we run separate sets of models for the three non-federal investment sources–using state and local government funding, nonprofit funding, and industry funding each as outcomes. Refer to Table 2 for the set of controls.

Tables 4, 5 and 6 present the results for each outcome–state and local, nonprofit, and industry R&D, respectively. We re-report the primary results from Eq 1 as a benchmark in Column 1 of each table. The relaxed model with the instruments for the set of non-federal funding sources, *Z*_*int*_, set as predetermined (Eq 4) is presented in Column 2; the pooled OLS with double lags of the logged dependent variable (Eq 3) is presented in Column 3; and the institution-field and year fixed effects model (Eq 2) is presented in Column 4 for each table. As with the primary set of models, all funding sources are estimated in log form.

**Table 4:** State & Local R&D Log Expenditure Regression Results, Additional Model Specifications.

Variables	(1)	(2)	(3)	(4)
	Primary Model	Alternate Model	Double Lag OLS	Fixed Effects
*Logged Funding Source*				
Federal	0.217***	0.226***	0.133***	0.293***
	(0.077)	(0.074)	(0.025)	(0.0483)
Industry	0.075**	0.060***	0.060***	0.0475***
	(0.036)	(0.015)	(0.008)	(0.00883)
Nonprofit	0.036**	0.036**	0.019**	0.0520***
	(0.015)	(0.015)	(0.007)	(0.00850)
Other	0.001	0.002	-0.006	0.00883
	(0.013)	(0.012)	(0.007)	(0.00770)
One Year Lagged Logged State & Local Funding	0.527***	0.527***	0.630***	
	(0.020)	(0.020)	(0.013)	
Two Year Lagged Logged State & Local Funding			0.167***	
			(0.013)	
Observations	13,840	13,840	10,380	17,300
Number of Institution-fields	3,460	3,460		3,460
Year Dummies Included	Yes	Yes	Yes	Yes
Institution-field Time Invariant Controls	Yes	Yes	No	Yes
*Post-specification Test Results*				
Serial Correlation AR(2) Pr > z	Fail to Pass	Fail to Pass		
Sargan/Over-Identification (Prob > chi2)	Fail to Pass	Fail to Pass		

*Notes*: Column 1 re-reports results from Eq 1 estimations (Table 3, Column 1); Clustered standard errors in parentheses (clustered by field);

*** p<0.01,

** p<0.05,

* p<0.1. All funding adjusted to FY 2009 and estimated in log form; dependent variable is logged state and local funding level; coefficients are elasticities. Columns 1 & 2, federal funding (predetermined) uses time t – l, where l≥1, to instrument for t; Columns 1 & 2, lagged dependent variable (endogenous) uses time t–k, where k ≥ 2, to instrument for t– 1; Column 1, other set of funding sources (endogenous) use time t–k, where k ≥ 2, to instrument for t; Column 2, other set of funding sources (predetermined) use time t – l, where l≥1, to instrument for t. Institution-field time invariant controls refers to first difference for Columns 1 and 2 and institution-field fixed effects for Column 4 (*α*_*in*_ from Eq 2).

**Table 5:** Nonprofit R&D Log Expenditure Regression Results, Additional Model Specifications.

Variables	(1)	(2)	(3)	(4)
	Primary Model	Alternate Model	Double Lag OLS	Fixed Effects
*Logged Funding Source*				
Federal	0.411***	0.471***	0.319***	0.271***
	(0.073)	(0.069)	(0.025)	(0.0482)
Industry	0.139***	0.036**	0.026***	0.0331***
	(0.036)	(0.015)	(0.008)	(0.00882)
State & Local	0.045***	0.046***	0.019***	0.0518***
	(0.014)	(0.014)	(0.006)	(0.00848)
Other	0.033**	0.044***	0.019***	-0.0139*
	(0.013)	(0.013)	(0.007)	(0.00769)
One Year Lagged Logged Nonprofit Funding	0.460***	0.466***	0.578***	
	(0.018)	(0.018)	(0.012)	
Two Year Lagged Logged Nonprofit Funding			0.138***	
			(0.012)	
Observations	13,840	13,840	10,380	17,300
Number of Institution-fields	3,460	3,460		3,460
Year Dummies Included	Yes	Yes	Yes	Yes
Institution-field Time Invariant Controls	Yes	Yes	No	Yes
*Post-specification Test Results*				
Serial Correlation AR(2) Pr > z	Pass	Pass		
Sargan/Over-Identification (Prob > chi2)	Fail to Pass	Fail to Pass		

*Notes*: Column 1 re-reports results from Eq 1 estimations (Table 3, Column 2); Clustered standard errors in parentheses;

*** p<0.01,

** p<0.05,

* p<0.1. All funding adjusted to FY 2009 and estimated in log form; dependent variable is logged nonprofit funding level; coefficients are elasticities. Columns 1 & 2, federal funding (predetermined) uses time t – l, where l≥1, to instrument for t; Columns 1 & 2, lagged dependent variable (endogenous) uses time t–k, where k ≥ 2, to instrument for t– 1; Column 1, other set of funding sources (endogenous) use time t–k, where k ≥ 2, to instrument for t; Column 2, other set of funding sources (predetermined) use time t – l, where l≥1, to instrument for t. Institution-field time invariant controls refers to first difference for Columns 1 and 2 and institution-field fixed effects for Column 4 (*α*_*in*_ from Eq 2).

**Table 6:** Industry R&D Log Expenditure Regression Results, Additional Model Specifications.

Variables	(1)	(2)	(3)	(4)
	Primary Model	Alternate Model	Double Lag OLS	Fixed Effects
*Logged Funding Source*				
Federal	0.468***	0.461***	0.369***	0.340***
	(0.076)	(0.075)	(0.025)	(0.0464)
State & Local	0.006	0.026*	0.054***	0.0440***
	(0.030)	(0.015)	(0.007)	(0.00817)
Nonprofit	0.031**	0.031**	0.023***	0.0307***
	(0.015)	(0.015)	(0.008)	(0.00819)
Other	0.069***	0.068***	0.026***	0.0247***
	(0.013)	(0.013)	(0.007)	(0.00741)
One Year Lagged Logged Industry Funding	0.428***	0.427***	0.549***	
	(0.019)	(0.019)	(0.013)	
Two Year Lagged Logged Industry Funding			0.178***	
			(0.013)	
Observations	13,840	13,840	10,380	17,300
Number of Institution-fields	3,460	3,460		3,460
Year Dummies Included	Yes	Yes	Yes	Yes
Institution-field Time Invariant Controls	Yes	Yes	No	Yes
*Post-specification Test Results*				
Serial Correlation AR(2) Pr > z	Pass	Pass		
Sargan/Over-Identification (Prob > chi2)	Fail to Pass	Fail to Pass		

*Notes*: Column 1 re-reports results from Eq 1 estimations (Table 3, Column 3); Clustered standard errors in parentheses (clustered by field);

*** p<0.01,

** p<0.05,

* p<0.1. All funding adjusted to FY 2009 and estimated in log form; dependent variable is logged industry funding level; coefficients are elasticities. Columns 1 & 2, federal funding (predetermined) uses time t – l, where l≥1, to instrument for t; Columns 1 & 2, lagged dependent variable (endogenous) uses time t–k, where k ≥ 2, to instrument for t– 1; Column 1, other set of funding sources (endogenous) use time t–k, where k ≥ 2, to instrument for t; Column 2, other set of funding sources (predetermined) use time t – l, where l≥1, to instrument for t. Institution-field time invariant controls refers to first difference for Columns 1 and 2 and institution-field fixed effects for Column 4 (*α*_*in*_ from Eq 2).

The results for the primary explanatory variable–federal R&D–are robust across all of the additional model specifications for the three outcomes. The size of the effect of federal R&D is fairly consistent but is slightly larger in the dynamic panel models for the outcomes of nonprofit and industry funding. With a few exceptions, the results for the additional non-federal funding relationships are also efficient and consistent.

As another effort to examine the robustness of the results, we compare the empirical results from our primary model (Eq 1) against prior studies. Considerable scholastic attention has been placed on this relationship; however, these studies used alternative econometric methods and units of analysis [1, 3–4, 20–22]. Notably, we find results are sensitive to the time period and sample restrictions under consideration, yet overall there is also evidence of a complementary effect. Diamond [22] found that a $1 increase in federal spending on basic research led, on average, to a $0.62 increase in industry funding. Blume-Kohout, Kumar, and Sood [3] more recently relied on a more comprehensive R&D source for the outcome and found that a $1 increase in federal funding on average leads to a $0.26 increase in non-federal academic life sciences funding.

### Stratification Results

#### Academic division stratification

We expand our analysis by running our primary model on a series of sample stratifications to more appropriately understand how the effect of federal funding on a series of non-federal sources varies in different contexts [3]. We first stratify the sample by academic division to account for disciplinary differences [23–24] and to exploit the funding variation presented in Fig 1. Analysis by this more granular level–in contrast to the institution–is useful given that “disciplines have their own qualities, cultures, codes of conduct, values, and distinctive intellectual tasks” [25] (pg. 386). The results from this analysis show which academic fields are driving the overall effect of federal funding.

**Fig 2:**
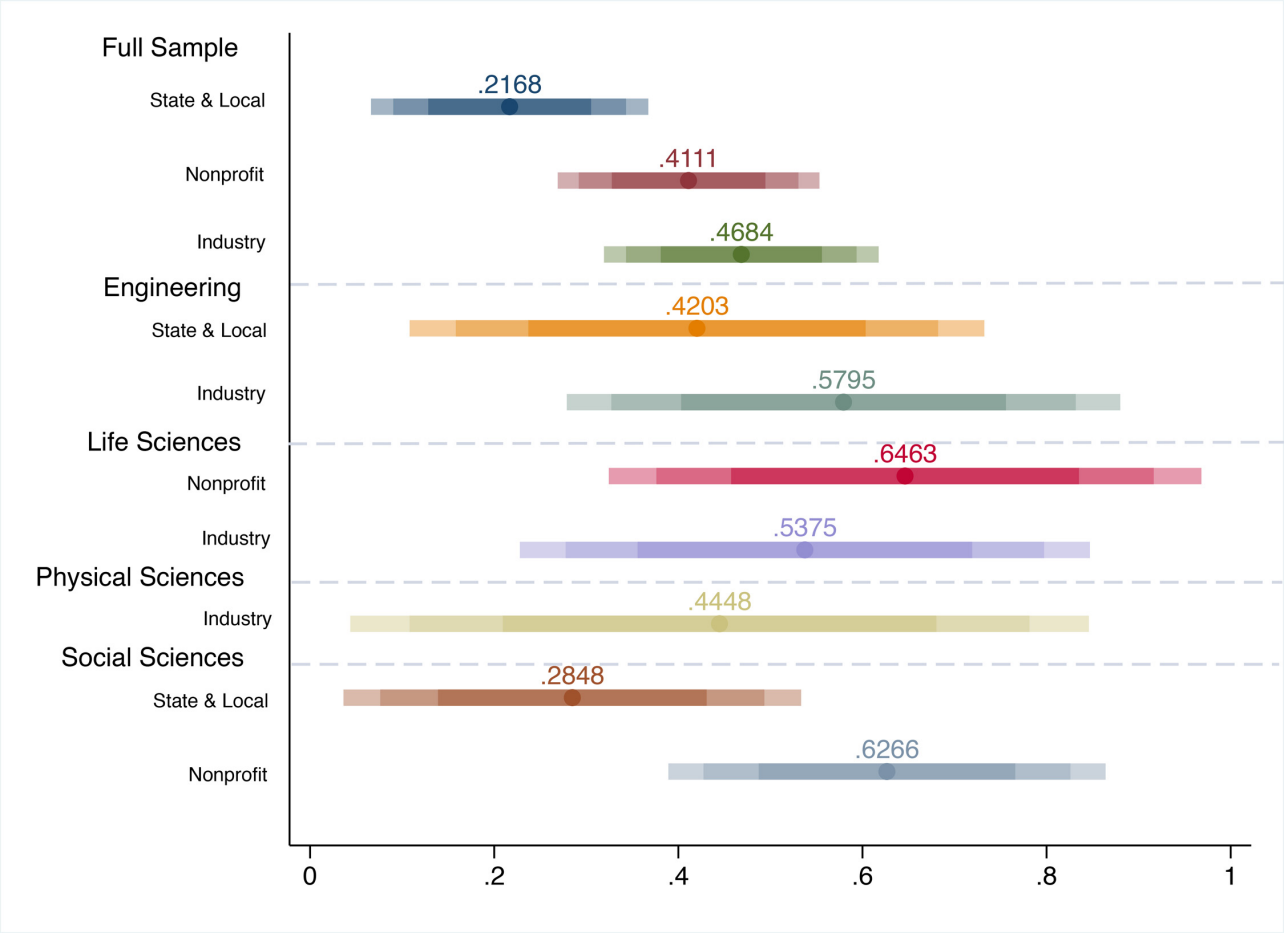
Robust Federal R&D Elasticity Confidence Intervals by Broad Academic Field and Non-Federal Source of Funding. *Notes*: Confidence intervals of elasticities from Eq 1 estimations are presented along the x-axis (95%, 90%, and 75%). Elasticity values are presented only if the results for the primary outcome–federal R&D funding–are efficient and consistent to the following additional model specifications: the alternate dynamic panel model (Eq 4), and the fixed effects model (Eq 2). The y-axis reports the sample stratifications with the respective outcome listed for each confidence interval. The dashed horizontal lines delimit the stratifications.

Fig 2 reports the confidence intervals for the robust results for academic divisions by outcome; the academic division stratifications are delimited by the dashed horizontal lines and the non-federal funding outcome is listed in line with the confidence interval on the y-axis. The results are robust only if they are statistically significant in the primary model (Eq 1) and efficient and consistent to the alternate dynamic panel model (Eq 4); and fixed effects model (Eq 2).

Fig 2 shows that while the elasticity of federal R&D funding is positive and significant for each outcome, significance is lost when stratified by division for certain fields. Specifically, we do not find an effect of federal funding for any outcome in the fields of mathematical and computer science or environmental science. However, we do find that for state and local funding the overall effect is being driven by the fields of engineering and social sciences and psychology with elasticities of 0.420 and 0.285, respectively. Alternately, for nonprofit funding, federal funding crowds in for the fields of social sciences and psychology and the life sciences such that a 1% increase in federal R&D funding is associated with an increase in nonprofit R&D funding by 0.627% in social sciences and psychology fields and by 0.646% in the life sciences. For industry, federal funding crowds in additional industry funding in the physical sciences (0.445), life sciences (0.537), and engineering (0.579).

#### Research capacity stratification

Funding from non-federal sources may be affected by the capacity to conduct research within a specific academic field. Certain scientific fields are notable regardless of characteristics of their institution. We include an additional stratification based on research capacity in each of the 26 academic scientific fields. We define *high research capacity* as the top quartile based on the distribution of total research expenditures for each respective academic field. We classify a field as being high capacity if at any point in the five-year time frame (2010–2014) they appear in the top quartile. All others are classified as low capacity.

**Fig 3:**
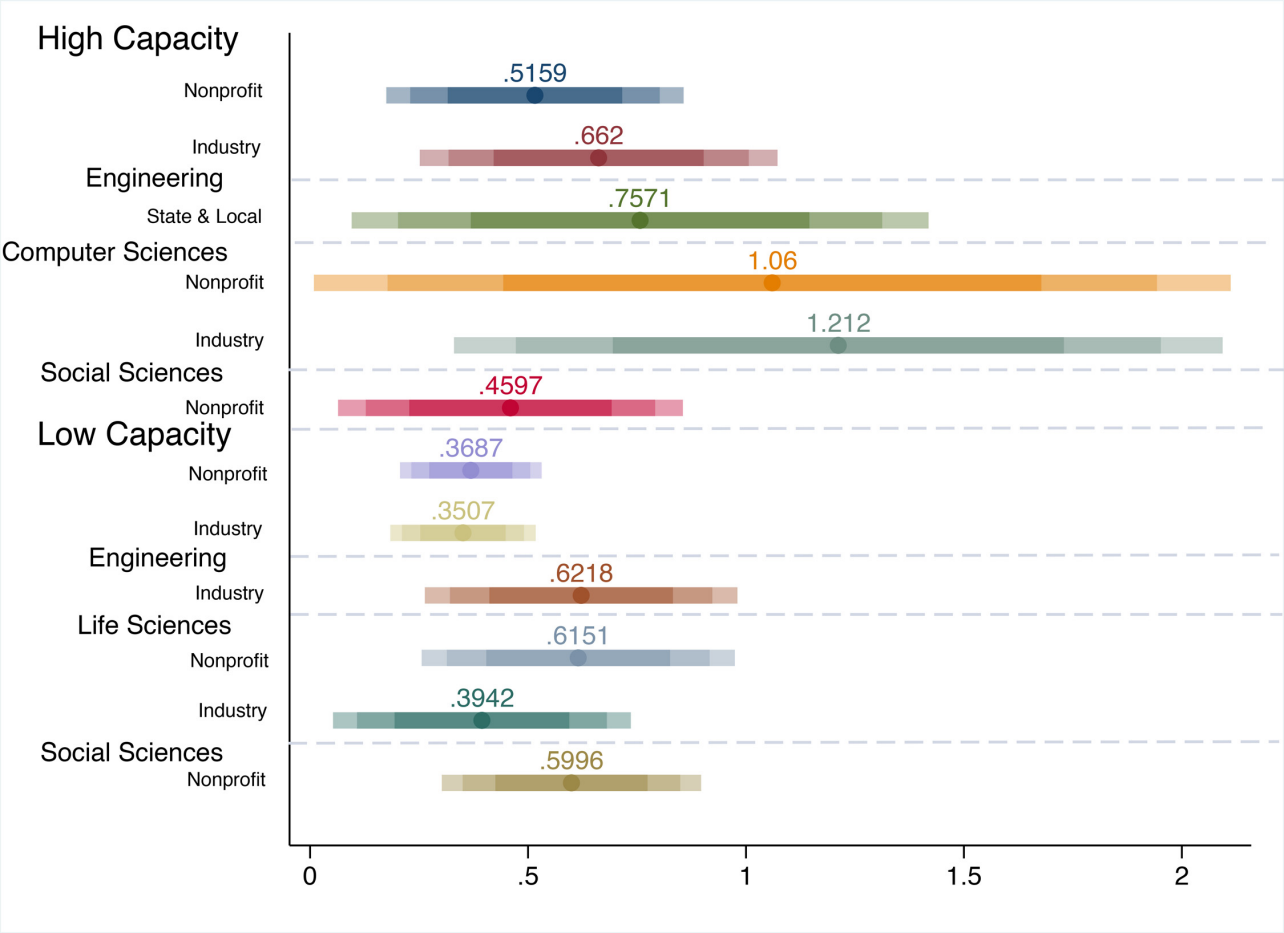
Robust Federal R&D Elasticity Confidence Intervals by Field Research Capacity, Broad Academic Division, and Non-Federal Source of Funding. *Notes*: Confidence intervals of elasticities from Eq 1 estimations are presented along the x-axis (95%, 90%, and 75%). Elasticity values are presented only if the results for the primary outcome–federal R&D funding–are efficient and consistent to the following additional model specifications: the alternate dynamic panel model (Eq 4), and the fixed effects model (Eq 2). The y-axis reports the sample stratifications with the respective outcome listed for each confidence interval. The dashed horizontal lines delimit the stratifications.

Fig 3 provides confidence intervals for the robust results for both high capacity and low capacity divisions. Again, the results are robust only if they are efficient and consistent to the following model specifications: (i) primary model (Eq 1); (ii) alternate dynamic panel model (Eq 4); and fixed effects model (Eq 2). The results are quite varied by outcome, division, and research capacity. For state and local funding, there is only evidence of federal crowd-in for high capacity engineering fields. The effect is much stronger with an elasticity of 0.757 compared to the elasticity of 0.420 for the full sample of engineering fields in Fig 2.

Regarding both nonprofit and industry funding, there are many more robustly significant findings. For the full sample, there is evidence of federal crowd-in of both industry and nonprofit funding for both high and low capacity fields, but the effect is larger for high capacity fields. The results straddle the elasticities of Fig 2. For industry funding, the higher crowd-in rate in high capacity fields is being driven by high capacity fields from the mathematical and computer science division (denoted in the figure as Computer Sciences), with an elasticity of 1.212. For nonprofits, the effect is also concentrated in the mathematical and computer science fields with an elasticity of 1.060 with an additional smaller effect in social sciences and psychology of 0.460. The elasticities for high capacity mathematical and computer sciences are the largest with over a 1:1 return on federal funding; this points to a greater responsiveness to marginal increases in federal funding activity.

Regarding low capacity findings, the results from Fig 3 for nonprofits and industry are similar to those in Fig 2. For industry funding, low capacity life science and engineering divisions exhibit federal crowd-in with elasticities of 0.394 and 0.622, respectively. This may reflect industry preference for working locally or working for academic units with a more applied orientation [26]. For nonprofit funding, social sciences and psychology and life sciences low capacity fields also show signs of federal crowd-in with elasticities of 0.600 and 0.615, respectively. This may reflect the nonprofit’s aim to build capacity and address under-researched topics.

#### Institutional control stratification

One distinguishing characteristic of U.S. research universities is the form of institutional governance. Universities may operate with a state mandate, providing some portion of public control over their operations, or they may be entirely private entities. Private sector governance with its greater autonomy may result in stronger performance. Aghion et al. [27] find evidence supportive of this claim in their study of university governance structures on academic research output as measured by institutional rankings and patents. However, a related study examining the effects of research spending on knowledge production by university control type does not find substantively different effects in follow-on funding between public and private universities [28]. While the results from their analysis reveal preliminary patterns of differences between these two governance structures, the authors indicate that further work is needed on this topic.

**Fig 4:**
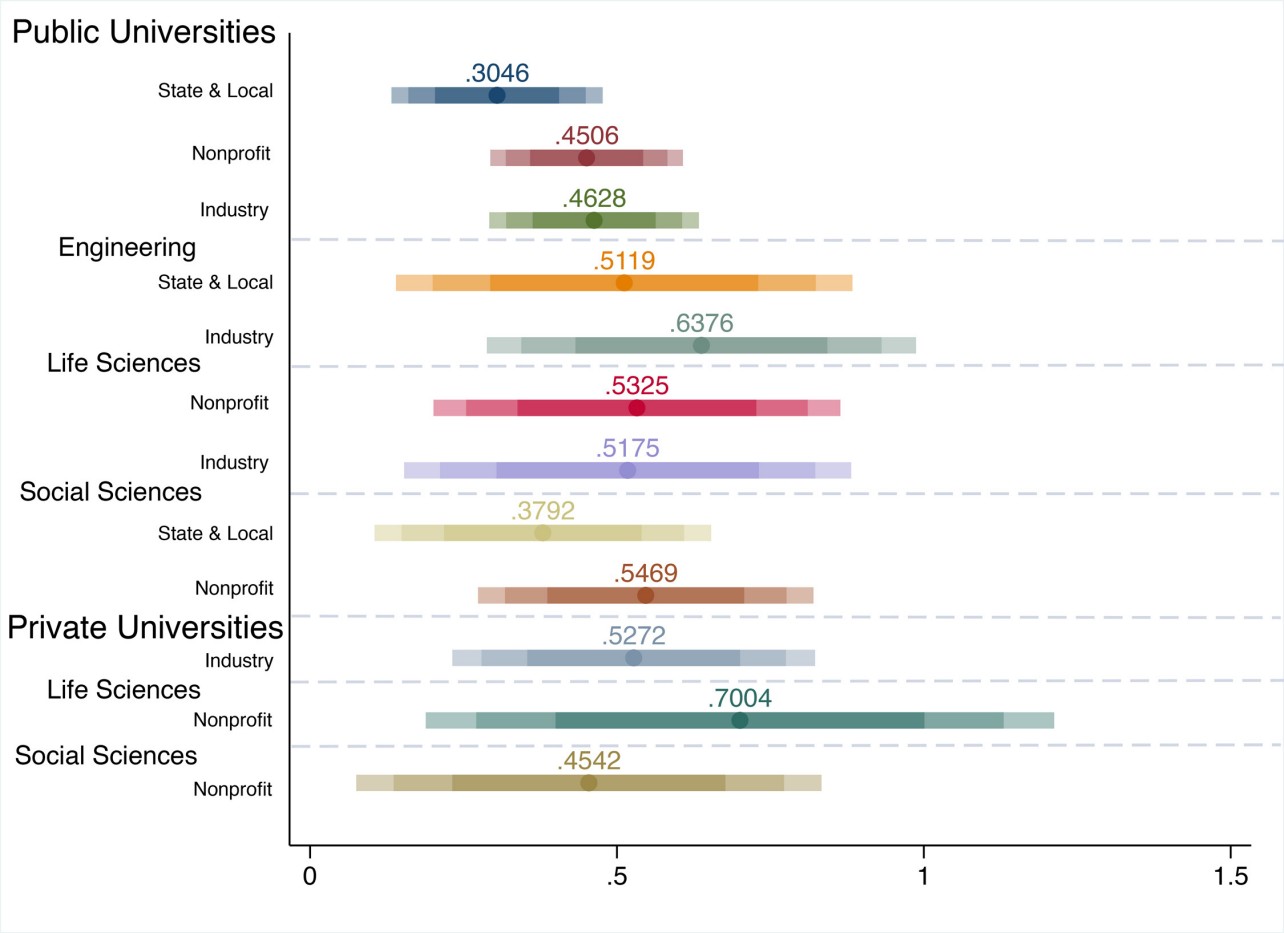
Robust Federal R&D Elasticity Confidence Intervals by Institutional Control, Broad Academic Division, and Non-Federal Source of Funding. *Notes*: Confidence intervals of elasticities from Eq 1 estimations are presented along the x-axis (95%, 90%, and 75%). Elasticity values are presented only if the results for the primary outcome–federal R&D funding–are efficient and consistent to the following additional model specifications: the alternate dynamic panel model (Eq 4), and the fixed effects model (Eq 2). The y-axis reports the sample stratifications with the respective outcome listed for each confidence interval. The dashed horizontal lines delimit the stratifications.

Following these previous analyses, we stratify the academic divisions by institutional control, either public or private. Seventy-three percent of the academic fields in the sample are part of public institutions. Fig 4 presents the confidence intervals for the robust results of the academic division by institutional type for the respective non-federal funding source. We use the same procedure as the two stratifications presented above to determine the set of robust results.

Given that a large share of the fields in the sample are part of public universities, it is not surprising that the robust results for the public university stratifications resemble the results presented in Fig 2. The similarities are still present when stratified by division for public universities with state and local funding showing evidence of federal crowd-in for social sciences and psychology and engineering fields, nonprofits in social sciences and psychology and life sciences, and industry for life sciences and engineering, all of which are also very similar in size to the generalized findings in Fig 2.

For private universities, where we find a robust effect, the effect size is larger. There is evidence of federal crowd-in of industry funding with an elasticity of 0.527. For nonprofit funding, a 1% increase in federal funding is associated with an increase in nonprofit funding by 0.454% in social sciences and psychology and by 0.7% in the life sciences for private universities.

**Fig 5:**
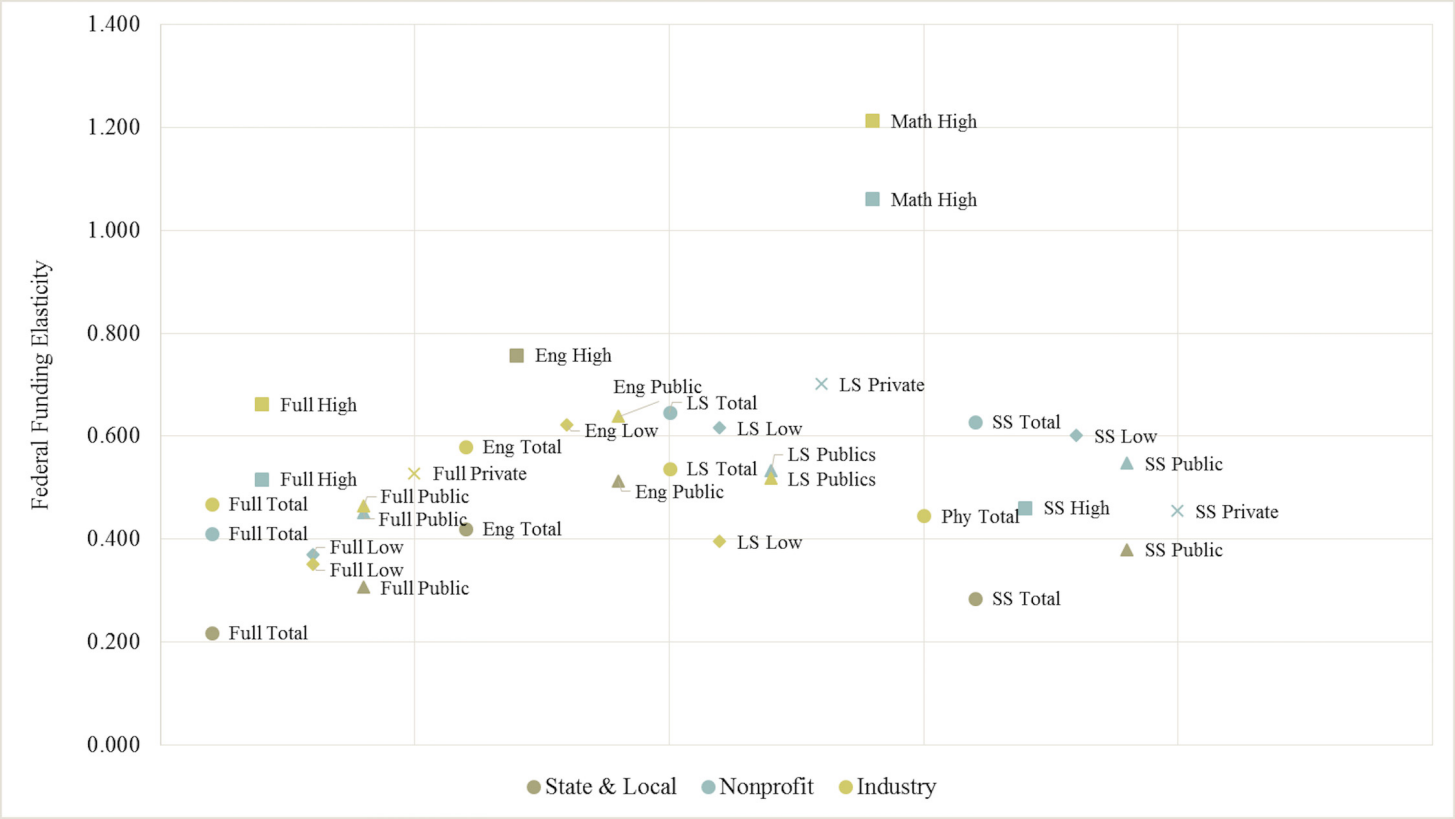
Robust Significant Federal Elasticities by Outcome & Stratification. *Notes*: Elasticities from Eq 1 estimations. Elasticity values are presented only if the results for the primary outcome–federal R&D funding–are efficient and consistent to the following additional model specifications: the alternate dynamic panel model (Eq 4), and the fixed effects model (Eq 2). The key for Fig 5 is presented directly above. The y-axis denotes the elasticity values, while the x-axis does not indicate any value but rather allows for the presentation of the myriad results. Additionally, Table 7 serves as a reference guide for the output in Fig 5. The left column denotes the abbreviations for the Fields and the right column references the various stratification and its corresponding referenced object in Fig 5.

Taking this series of stratified results together, Fig 5 presents all of the robust elasticities by model specification across the three stratifications by outcome and sample. Excluding the large elasticities of high capacity mathematical and computer science fields, all of the elasticities fall between 0.200 and 0.800. On average, state and local funding tends to exhibit slightly lower federal elasticities compared to nonprofit and industry funding.

**Table 7:** Field and Stratification Abbreviations for Fig 5.

Fields	Stratifications
**Full**—Full Sample	**Total** Sample delimited by a circle
**Eng**–Engineering	**High** Research Capacity delimited by a square
**LS**—Life Sciences	**Low** Research Capacity delimited by a diamond
**Phy**—Physical Sciences	**Public** University delimited by a triangle
**SS**—Social Sciences & Psychology	**Private** University delimited by an X
**Math**—Mathematical & Computer Sciences	

### Additional Results and Empirical Considerations

#### Robust to additional model specifications vs. statistically significant results for primary model (Eq 1)

For each of the Figs 2, 3, 4 and 5, elasticities are presented only if the results for the primary outcome–federal R&D–are robust. Whereby robust means the elasticities are statistically significant and consistent across multiple model specifications of: (i) the primary model (Eq 1); (ii) the alternate dynamic panel model (Eq 4); and (iii) the fixed effects model (Eq 2). To present the greater range of results for the primary estimation (Eq 1), Table 8 presents both the robust and non-robust results. The non-robust are still statistically significant federal elasticities to the Eq 1 estimation, yet not consistent and efficient across the set of additional model specifications. The table includes the federal funding elasticity and standard error for each stratified regression by outcome. Thus each coefficient is from a unique regression. The black cells are robust federal elasticities and the black bold cells are those federal elasticities that are statistically significant under the primary estimation (Eq 1), but are not robust the two additional model specifications. The results presented in black bold indicate more activity in the fields of environmental sciences, mathematical and computer sciences, and the physical sciences.

**Table 8:** Comparison of Significant (Eq 1) and Robust Federal Funding Elasticities by Stratification.

Sample	(1)	(2)	(3)	Sample Size
	State & Local	Nonprofit	Industry	
Full Sample	0.217***	0.411***	0.468***	13,840
	(0.077)	(0.073)	(0.076)	
*By Broad Discipline*				
Engineering	0.420***		0.579***	3,684
	(0.159)		(0.153)	
Environmental Sciences			0.511**	1,376
			**(0.213)**	
Life Sciences		0.646***	0.537***	2,480
		(0.164)	(0.158)	
Mathematics & Computer Science			0.869***	1,536
			**(0.227)**	
Physical Sciences			0.445**	2,068
			(0.205)	
Psychology & Social Sciences	0.285**	0.627***		2,696
	(0.127)	(0.121)		
*By Broad Discipline & Funding Capacity*				
Full Sample, High Capacity		0.516***	0.662***	4,264
		(0.174)	(0.209)	
Full Sample, Low Capacity		0.369***	0.351***	9,576
		(0.083)	(0.085)	
Engineering, High Capacity	0.757**			1,188
	(0.338)			
Engineering, Low Capacity			0.622***	2,496
			(0.183)	
Life Sciences, High Capacity		0.621**		688
		**(0.317)**		
Life Sciences, Low Capacity		0.615***	0.394**	1,792
		(0.183)	(0.174)	
Mathematics & Computer Science, High Capacity		1.060**	1.212***	476
		(0.537)	(0.450)	
Mathematics & Computer Science, Low Capacity			0.666**	1,060
			**(0.271)**	
Psychology & Social Sciences, High Capacity		0.460**		884
		(0.202)		
Psychology & Social Sciences, Low Capacity		0.600***		1,812
		(0.152)		
*By Broad Discipline & University Type*				
Full Sample, Public	0.305***	0.451***	0.463***	10,068
	(0.088)	(0.080)	(0.087)	
Engineering, Public	0.512***		0.638***	2,684
	(0.190)		(0.178)	
Life Sciences, Public		0.533***	0.517***	1,836
		(0.169)	(0.186)	
Physical Sciences, Public			0.633***	1,452
			**(0.228)**	
Psychology & Social Sciences, Public	0.379***	0.547***		1,948
	(0.140)	(0.139)		
Environmental Sciences, Public			0.561**	1,072
			**(0.229)**	
Math & Computer Sciences, Public			0.845***	1,076
			**(0.242)**	
Full Sample, Private			0.527***	3,772
			(0.151)	
Math & Computer Sciences, Private		0.810***		460
		**(0.245)**		
Life Sciences, Private		0.700***	0.635***	644
		(0.261)	**(0.237)**	
Psychology & Social Sciences, Private		0.454**		748
		(0.193)		

*Notes*: Column 1 presents results with state and local R&D as the outcome (referring to Equations A and A.1 – A.5 in supplementary materials–S3 File); Column 2 presents results with nonprofit R&D as the outcome (referring to Equations B and B.1 – B.5 in supplementary materials–S3 File); Column 3 presents results with industry R&D as the outcome (referring to Equations C and C.1 – C.5 in supplementary materials–S3 File); Coefficients and standard errors are reported; coefficients represent elasticities; clustered standard errors (clustered by institution-field);

*** p<0.01,

** p<0.05,

* p<0.1. Each elasticity is the federal funding coefficient by outcome across stratifications. Each elasticity is from a different regression estimating Eq 1 on the sub-sample of the specified stratification and outcome. Black cells are those elasticities that are significant and robust to a series of model specifications: (i) the primary model (Eq 1); (ii) the alternate dynamic panel model (Eq 4); and the fixed effects model (Eq 2). **Black bold** cells are statistically significant elasticities (Eq 1), but are not robust to the full series of model specifications.

#### Sensitivity assessment of instrument specification for dynamic panel model

We rely on a dynamic panel model as the primary model (Eq 1) to account for both the prior activity of the dependent variable and time-invariant institution-field factors. At the same time, we are constrained with having institution-field level funding data over a five-year time frame. To illustrate the sensitivity of the results for this empirical approach, Table 9 presents the statistically significant federal elasticities both from the primary model (Eq 1) and the alternate dynamic panel model (Eq 4). The latter relaxes the assumption for the instrument specification on the vector of additional non-federal variables from endogenous to predetermined. As in Table 8, each elasticity presented is the response to the federal funding and comes from a unique regression by outcome and stratification. Elasticities presented in Columns 1, 3, and 5 are from the primary model (Eq 1) and specify the non-federal funding controls as endogenous, while the elasticities presented in Columns 2, 4, and 6, are from the alternate model (Eq 4) and define the non-federal controls as predetermined. The results across the two models are very similar, though the range of significant results is greater for the alternate dynamic panel model (Eq 4) with the relaxed assumption (as we would expect). This illustrates the sensitivity of the results due to the instrument specification.

**Table 9:** Comparison of Endogenous vs. Predetermined Controls across Outcomes and Stratifications (Eq 1 & Eq 4).

	State & Local		Nonprofit		Industry		Sample Size
Sample	(1)	(2)	(3)	(4)	(5)	(6)	
	Primary	Alternate	Primary	Alternate	Primary	Alternate	
Full Sample	0.217***	0.226***	0.411***	0.471***	0.468***	0.461***	13,840
	(0.077)	(0.074)	(0.073)	(0.069)	(0.076)	(0.075)	
*By Broad Discipline*							
Engineering	0.420***	0.425***			0.579***	0.598***	3,684
	(0.159)	(0.154)			(0.153)	(0.152)	
Environmental Sciences					0.511**	0.476**	1,376
					(0.213)	(0.210)	
Life Sciences			0.646***	0.684***	0.537***	0.534***	2,480
			(0.164)	(0.150)	(0.158)	(0.153)	
Math & Computer Science				0.476***	0.869***	0.945***	1,536
				(0.184)	(0.227)	(0.217)	
Physical Sciences				0.373**	0.445**	0.416**	2,068
				(0.168)	(0.205)	(0.201)	
Psychology & Social Sciences	0.285**	0.294**	0.627***	0.629***			2,696
	(0.127)	(0.125)	(0.121)	(0.121)			
*By Broad Discipline & Funding Capacity*							
Full Sample, High Capacity			0.516***	0.525***	0.662***	0.631***	4,264
			(0.174)	(0.167)	(0.209)	(0.209)	
Full Sample, Low Capacity			0.369***	0.413***	0.351***	0.349***	9,576
			(0.083)	(0.080)	(0.085)	(0.084)	
Engineering, High Capacity	0.757**						1,188
	(0.338)						
Engineering, Low Capacity					0.622***	0.644***	2,496
					(0.183)	(0.183)	
Life Sciences, High Capacity			0.621**	0.613**			688
			(0.317)	(0.309)			
Life Sciences, Low Capacity			0.615***	0.658***	0.394**	0.411**	1,792
			(0.183)	(0.175)	(0.174)	(0.172)	
Math & Computer Science, High Capacity			1.060**	0.962**	1.212***	1.138***	476
			(0.537)	(0.481)	(0.450)	(0.425)	
Math & Computer Science, Low Capacity					0.666**	0.687**	1,060
					(0.271)	(0.268)	
Physical Sciences, High Capacity		-0.898**					600
		(0.442)					
Psychology & Social Sciences, High Capacity			0.460**	0.446**			884
			(0.202)	(0.198)			
Psychology & Social Sciences, Low Capacity			0.600***	0.592***			1,812
			(0.152)	(0.151)			
*By Broad Discipline & University Type*							
Full Sample, Public	0.305***	0.306***	0.451***	0.501***	0.463***	0.458***	10,068
	(0.088)	(0.084)	(0.080)	(0.076)	(0.087)	(0.084)	
Engineering, Public	0.512***	0.481***		0.494***	0.638***	0.668***	2,684
	(0.190)	(0.177)		(0.165)	(0.178)	(0.175)	
Life Sciences, Public			0.533***	0.585***	0.517***	0.515***	1,836
			(0.169)	(0.157)	(0.186)	(0.182)	
Physical Sciences, Public				0.427**	0.633***	0.592***	1,452
				(0.213)	(0.228)	(0.215)	
Psychology & Social Sciences, Public	0.379***	0.390***	0.547***	0.542***			1,948
	(0.140)	(0.140)	(0.139)	(0.140)			
Environmental Sciences, Public					0.561**	0.614***	1,072
					(0.229)	(0.234)	
Math & Computer Sciences, Public				0.465**	0.845***	0.913***	1,076
				(0.216)	(0.242)	(0.233)	
Full Sample, Private				0.344**	0.527***	0.515***	3,772
				(0.140)	(0.151)	(0.149)	
Math & Computer Sciences, Private			0.810***	0.827***			460
			(0.245)	(0.246)			
Life Sciences, Private			0.700***	0.734***	0.635***	0.683***	644
			(0.261)	(0.230)	(0.237)	(0.229)	
Psychology & Social Sciences, Private			0.454**	0.467**			748
			(0.193)	(0.196)			

*Notes*: Coefficients and standard errors are reported; coefficients represent elasticities; clustered standard errors (clustered by institution-field);

*** p<0.01,

** p<0.05,

* p<0.1. Each elasticity is the federal funding coefficient by outcome across stratifications. Each elasticity is from a different regression run on the sub-sample of the specified stratification and outcome. Elasticities from the primary model, columns 1, 3, and 5, specify the controls of non-federal funding as *endogenous* (Eq 1) and uses time t–k, where k ≥ 2, to instrument for t. Elasticities from the alternate model, columns 2, 4, and 6, specify the controls of non-federal funding as *predetermined* (Eq 4) and uses time t – l, where l≥1, to instrument for t.

### Policy Significance

The statistically significant findings from this analysis are of policy significance. In this section we provide interpretations of the robust elasticities based on the mean calculations for the various academic fields–which again is interpreted as analogous to academic departments. To calculate these examples, the average funding levels were pulled from the various stratifications by federal, nonprofit, state and local, and industry sources. The descriptive statistics on the mean distributions, by source and stratification, are provided in S1 Table. Again, the elasticity is interpreted as a 1% change in federal funding being associated with a Beta% change in the funding level of nonprofit, state and local, or industry.

Consider the case of the division of life sciences. Academic fields in the life sciences at public universities have an average federal investment of approximately $22 million. A 1% increase in federal funding of approximately $224,000, is associated with a 0.517% increase in industry funding and a 0.533% increase in nonprofit funding. Given the average industry funding level of $1.7 million and $2.4 million nonprofit funding, this marginal increase in federal funding would be associated with a crowding in of an additional $8,900 from industry and $12,800 from nonprofits. Meanwhile, the life sciences at private universities are operating with much larger budgets, on average, with average federal funding of $43 million and $5 million in nonprofit funding. A 1% increase in federal funding of approximately $433,000 is associated with a 0.7% increase in nonprofit funding of approximately $35,000, on average.

To contrast, engineering fields operate with significantly smaller budgets than life sciences. Even high capacity engineering fields have an average federal funding level of approximately $15 million. A 1% increase in their federal funding of $150,000 is associated with a 0.757% increase in state and local funding. With an average state and local budget of $1.3 million, the federal increase would crowd in an additional $9,500 from state and local sources. However, a low capacity engineering field has an average federal funding level of just $1.8 million; so a 1% increase in federal funding would be an additional $18,000. Based on our findings, this would crowd in industry funding. Given the average industry funding level of $222,000, this would lead to an additional $1,400 in industry funding.

Similarly, the social sciences and psychology fields have smaller budgets on average, ranging from an average of $609,000 for low capacity fields to $5.4 million for high capacity. Each exhibits a positive crowd in from nonprofit funding with elasticities of 0.460% and 0.6% from a 1% increase in federal funding. This would provide an additional $4,000 to high capacity fields and $600 to low capacity fields given the average nonprofit funding levels of $875,000 and $100,000 respectively.

As seen from these illustrations, the results from this analysis find consistent crowding in from federal funding but the impact varies across field characteristics. While the robust elasticities are positive, the majority of the coefficients are less than one. However, industry and nonprofit funding from high research capacity math and computer science fields report robust elasticities greater than one, pointing to a higher level of responsiveness to changes in federal R&D funding.

## Conclusion

Investments in science provide knowledge and discoveries that advance national priorities and drive economic growth. Our analysis demonstrates a complementary relationship between federal science funding and non-federal funders. Each funder of academic science has unique objectives, which include commercial success for private industry, societal benefit for nonprofit organizations, and local economic development for state and local governments. The results suggest that the organizations that fund science are all part of a system that is positively influenced by federal research investment activity. Rather than crowd out additional investment, we find evidence that federal science funding crowds in additional funding from industry, nonprofits, and state and local governments, thereby furthering their objectives.

In this analysis, we focus at the level of academic science fields–analogous to academic departments–within the university context. Rather than estimate the relationships at the aggregate institution level, we focus more closely on the unit of research production and draw upon the population of research-active scientific fields at U.S. doctoral granting research universities. Using instrumental variables, we include both the lagged logged dependent variable to account for prior levels of the non-federal funding source and a set of first differences R&D funding regressors to control for time-invariant factors that may account for the institution-field’s ability to secure funding. Using the NSF HERD Survey, we draw upon a broader portfolio of funding sources than previously available. We examine nonprofit organizations using new data that have not previously been available. The analysis is limited to consider the five years for which data are currently available; however the analysis should be expanded in the future as more years become available.

While other countries are increasing their commitment to funding science, U.S. science suffers under Congressional control of discretionary funding [29]. Current funding levels from non-federal sources are not able to compensate for decreases in federal research funding. Moreover–as the results from this analysis suggest–decreases in federal research funding would likely be associated with a decrease in research investment from other sources. From a more positive perspective, federal research funding is not only a large source of investment for academic science; it also induces investment from myriad other sources.

## Supporting Information

S1 File Data Building. (DOCX)

S2 File Technical Note for Estimation Method. (DOCX)

S3 File Detailed Notation. (DOCX)

S1 Table Descriptive Statistics for Elasticity Computation. (DOCX)
