# Supplementary material for: The Domino Effects of Federal Research Funding
Source: PLoS One. 2016 Jun 21;11(6):e0157325. doi: 10.1371/journal.pone.0157325 (PMC4915724; doi:10.1371/journal.pone.0157325)
Supplement: S1 File — (DOCX) [file pone.0157325.s001.docx]

# S1 File. Data Building.

All data used for this project are publicly available and accessible online. We have annotated the entire data building process and empirical techniques presented in the paper. We have made the code for both components – data building and empirical analysis – publicly available online; we relied on STATA for these components. In addition, we have uploaded the cleaned dataset. This is available at: doi:10.7264/N3W957G6. This is accessible at the following webpage: <http://hdl.handle.net/1794/19409>. Below we outline our approach for building the dataset from the raw public files.

Data on U.S. academic field R&D funding was downloaded from the National Science Foundation (NSF) Higher Education Research and Development (HERD) Survey. U.S. university field level data was pulled from the file R&D Expenditures, By Source of Funds (2010 – 2014) for the following sources of funding: Federally Financed R&D Expenditures, Business Financed R&D Expenditures (Industry), State/Local Govt. Financed R&D Expenditures, Nonprofit Financed R&D Expenditures, Institutionally Financed R&D Expenditures (Own Institutional), and R&D Expenditures funded by All Other Sources (including foreign). The NSF HERD Survey provides detailed data on 36 unique fields that are standardized across institutions; these include: aerospace engineering, agricultural sciences, arts and music, astronomy, atmospheric sciences, biological sciences, business and management, chemical engineering, chemistry, civil engineering, communication and librarianship, computer science, earth science, economics, education, electrical engineering, humanities, interdisciplinary or other sciences, law, materials engineering, mathematics and statistics, mechanical engineering, medical sciences, not available, oceanography, other engineering, other geosciences, other life sciences, other non-sciences or unknown disciplines, other physical sciences, other social sciences, physics, political science and public administration, psychology, social service professions, and sociology.

The full survey population is 122,688 field level observations for U.S. institutions that annually perform at least $150,000 in budgeted R&D within any field and have degree programs at the bachelor's level or higher.

The remainder of the section documents additional efforts to increase the level of detail and vet the accuracy of the NSF HERD survey. While, we did not directly draw upon these sources for the empirical analysis, these efforts confirmed our understanding of the scope, scale, and accuracy of the NSF HERD survey. First, we matched the data to the National Center for Education Statistics (NCES) Integrated Postsecondary Education Data System (IPEDS). We relied on a series of matching procedures based on a version of the string name of the university. We programmed a code to match 69.4% of the observations and then hand-matched the remaining observations using a common string match. The overall match rate for this exercise was 97.24%.

Next, we merged the database with university level data from the Delta Cost Project based on a unique IPEDS identification number. The Delta Cost data provides publicly accessible panel data on higher education finances, enrollment, staffing, completion, and student aid (university level). The match rate for this exercise was 91.3% of the sample with IPEDS id; this represents 86.3% of the overall NSF HERD sample. Among those that did not match with Delta Cost, we manually searched each institution’s research status with the National Center for Education Statistics (NCES) database. We coded the institutions that offer a “Doctor’s research/scholarship” degree. We argue that this approach includes an inclusive sample of higher education research-based institutions. We confirmed that those without a match for the sample of the population with an IPEDS id included educational institutions such as community colleges, beauty schools, training and fitness institutions, seminaries, or institutions granting “Doctor’s professional practice.”

For this analysis, we define a population of U.S. higher education science and engineering (S&E) fields with a research focus. Thus, we kept the university field observations from the NSF HERD survey based on the following stratifications: (i) demonstrated active Federal R&D funding portfolio over the five-year time frame (2010 – 2014); (ii) Doctoral-granting; and (iii) NSF HERD survey broad division classification – engineering, physical sciences, environmental sciences, mathematical sciences, computer sciences, life sciences, psychology, and social sciences. For the former, we included academic fields with a positive Federal R&D funding stream over the entirety of the panel (2010 – 2014). For the second, we defined “Doctoral-granting” by the NCSES measure – Highest Degree Granting. We excluded specialized institutions with a medical or engineering focus (as defined by the Carnegie classification reported via NSF’s WebCASPAR). For the latter stratification, we removed fields in the fields of humanities, professional programs, and interdisciplinary studies from the analysis.

Based on the distribution of fields in the sample, we then classified the following divisions into one broad division, respectively – mathematical sciences and computer sciences collapsed into “math and computer science” and social sciences and psychology collapsed into “social science and psychology.” The analysis is based on 26 science fields across the six division classifications (see crosswalk in Table 1 in the manuscript). The broad classifications are comprised of the following fields: Engineering: aerospace engineering, chemical engineering, civil engineering, electrical engineering, materials engineering, mechanical engineering, other engineering; Physical Sciences: astronomy, chemistry, other physical sciences, physics; Environmental Sciences: atmospheric sciences, earth sciences, oceanography, other geosciences; Mathematical Sciences and Computer Sciences: mathematics and statistics, computer science; Life Sciences: agricultural sciences, biological sciences, medical sciences, other life sciences; and Social Sciences and Psychology: psychology, economics, political science and public administration, sociology, other social sciences.

Based on these stratifications, we defined a sample of 3,460 unique field observations from 266 universities. On average, 13 fields are included for each institution (Minimum = 1; Maximum = 26; Standard Deviation = 6.53). Again, these fields have active federal R&D funding. Data are available for a five-year time frame, 2010 to 2014. The data are balanced; hence, the full dataset is based on 17,300 institution-fields over time.

We adjust funding levels to account for inflation using Gross Domestic Product Implicit Price Deflator, with 2009 as the base year and then use the natural log form in estimations. The NSF HERD Survey reports R&D funding data in $1,000s. We adjust this before taking the natural log. For field observations without non-Federal R&D funding, we recoded the level value to one such that the natural log was then equivalent to zero.
