# Supplementary material for: The Domino Effects of Federal Research Funding
Source: PLoS One. 2016 Jun 21;11(6):e0157325. doi: 10.1371/journal.pone.0157325 (PMC4915724; doi:10.1371/journal.pone.0157325)
Supplement: S2 File — (DOCX) [file pone.0157325.s002.docx]

# S2 File. Technical Note for Estimation Method.

This section provides an extended discussion of the Estimation Method presented in the manuscript. To reiterate, we are centrally interested in estimating the effect of federal R&D funding on a series of non-federal sources. Formally, we can express this relationship with the following function:

$Y_{int}=f(X_{int},Y_{int-1},Z_{int},A_{t}, \alpha_{in})$,

where *i* denotes the academic field, *n* indexes the institution, and *t* is the annual time period. *Y* is the outcome variable for the non-federal funding source of interest. We estimate effects for three outcomes: state and local, nonprofit, and industry R&D funding. *X* delimits the key explanatory variable – federal R&D funding. *Z* denotes the set of non-federal funding sources that excludes *Y* – the outcome variable being estimated. *A* captures annual general macroeconomic shocks that might affect R&D funding streams. $\alpha$ is an institution-field fixed effect to account for time-invariant institution-field factors. Lastly, we include the one-year lagged dependent variable, $Y_{t-1}$, to control for prior capacity to secure the non-federal funding outcome.

We are interested in the relationships between these different funding sources, which are endogenous and jointly determined. Inclusion of the one-year lagged dependent variable and fixed effects estimators alone, however, does not obviate endogeneity as the lagged component, $Y_{int-1}$, is correlated with the error component,$\varepsilon_{int-1}$, in the fixed effects model [1]. In their seminal paper, Arellano and Bond [2] offer a resolution by instrumenting the lagged dependent variable at least two periods in the fixed effects model. To increase the efficiency of the model, Blundell and Bond [3] developed an additional approach to instrument levels with differences rather than instrumenting differences (or orthogonal deviations) with levels [4]. This approach is valid under the assumption that the instrumenting variable, notated as *w,* is uncorrelated with the fixed effect – $E\left[ \Delta w_{int}\alpha_{in} \right]= 0$; in other words,$E\left[ w_{int}\alpha_{in} \right]$ is time-invariant [4] (pg. 28).

We draw upon these methods to include both first differences and the instrumented lagged dependent variable. In addition, dynamic panel models also utilize a set of instruments to account for endogeneity of prior trends of independent variables. For the primary explanatory variable, given that federal R&D funding has historically high and relatively stable levels of research investment [14], we treat this regressor as predetermined. This assumes that it is correlated with past errors, but uncorrelated with future errors. Federal funding is then instrumented with the following lags: $X_{int-1}$,$\ldots,X_{int-4}.$ [11, 13]. While this first lag may seem counterintuitive, Blundell and Bond [3] formalize this under the assumption of convergence between the fixed effect and lagged dependent variable.

Following this approach, the level of the lagged dependent variable from at least two prior time periods, $Y_{int-2},$ provides an instrument for the field’s capacity to secure the non-federal funding outcome: $\Delta Y_{int-1}=Y_{int-1}-Y_{int-2}$. Importantly, the second lag instrument, $Y_{int-2}$, and subsequent lags are not mathematically related with the second component of the error term, $\varepsilon_{int-1}$, where $\Delta\varepsilon_{int}=\varepsilon_{int}-\varepsilon_{int-1}$[4] (pg. 21). Taken together, this instrumental variables approach conditions on both first differences and the lagged dependent variable by addressing the endogeneity problem and accounts for the effect of spurious changes with additional contemporaneous non-federal funding sources.

To elaborate on the latter, we expect each of these funding sources to be influenced by federal funding levels and potentially to influence each other. Thus we include the portfolio of sources to account for spurious relationships. For example, changes in industry-funded research may influence federal funding investment for the field of engineering, causing a spurious correlation between nonprofit and federal funding. For the vector of non-federal regressors, $\Delta Z_{int}=Z_{int}-Z_{int-1}$, we estimate the model assuming that they are endogenous [6], where, $E\left( x_{int}\varepsilon_{int} \right)\neq0$. The vector with the one-year lag is not a valid instrument; hence, we instrument starting with the two year lag, $Z_{int-2}$, …, $Z_{int-4}$, for each source [6].

Equations A, B, and C, presented in the following Supplementary Section, S3 Detailed Notation – Model I Specification, are the primary models given that we are able to: (i) address endogeneity of the non-federal funding outcome variable by including the lag as a covariate on the right-hand side; (ii) include first differences to control for institution-field specific variation that otherwise would confound the results; and (iii) account for confounding factors that include other, contemporaneous non-federal funding activity.

As an alternative to first differencing, we considered using the National Research Council’s (NRC) survey on Research Doctorate Programs [7]. This survey is the most comprehensive program level data source (also eponymous to academic departments); however, it is decennial with the most recent round in 2005 – 2006, and thus slightly dated for the purposes of this sample. In addition, it represents roughly 30% of the NSF HERD sample with an active federal funding stream. This is attributed to limits with the scale of the NRC survey. Given this notable data constraint, we include the full sample from the NSF HERD data and rely on first differencing in the dynamic panel model to control for time-invariant factors.

### References for S2 File.

[1] Angrist JD, Pischke JS. Mostly harmless econometrics: An empiricist's companion. Princeton University Press; 2008 Dec 15.

[2] Arellano M, Bond S. Some tests of specification for panel data: Monte Carlo evidence and an application to employment equations. The Review of Economic Studies. 1991 Apr 1;58(2):277-97.

[3] Blundell R, Bond S. Initial conditions and moment restrictions in dynamic panel data models. Journal of Econometrics. 1998 Nov 30;87(1):115-43.

[4] Roodman D. How to do xtabond2: An introduction to difference and system GMM in Stata. Center for Global Development working paper. 2006 Dec(103).

[5] Historical trends in Federal R&D. 2014 Aug 14. AAAS R&D Budget and Policy Program. Available: http://www.aaas.org/page/historical-trends-federal-rd

[6] Cameron AC, Trivedi PK. Microeconometrics: methods and applications. Cambridge University Press; 2005 May 9.

[7] Ostriker J, Kuh CV, Voytuk JA. A Data-Based Assessment of Research-Doctorate Programs in the United States. The National Academies Press: Washington, DC. 2010.
