# Supplementary material for: The Domino Effects of Federal Research Funding
Source: PLoS One. 2016 Jun 21;11(6):e0157325. doi: 10.1371/journal.pone.0157325 (PMC4915724; doi:10.1371/journal.pone.0157325)
Supplement: S3 File — (DOCX) [file pone.0157325.s003.docx]

# S3 File. Detailed Notation.

We run four broad sets of model specifications: (i) a dynamic panel model that defines the vector of $Z_{int}$ as endogenous; (ii) institution-field and year fixed effects (Eq. 2); (iii) pooled OLS with the inclusion of two lagged logged dependent variables: $Y_{int-1}$ and $Y_{int-2}$ (Eq. 3) where the standard errors are clustered at the field level; and (iv) a dynamic panel model that defines the vector of $Z_{int}$ as predetermined rather than as endogenous. *All funding sources are estimated in log form for the models listed below.* All the equations rely on the follow indices: *i* denotes the field, *n* denotes the institution, and *t* denotes the year. The full notation for each set of models is detailed below for each outcome – state & local, nonprofit, and industry R&D.

**Model Specification I: Primary Dynamic Panel Model (Detailed Notation of Eq. 1)**

Equations A, B, and C present detailed notation for the primary dynamic panel model for the three outcomes: state and local, nonprofit, and industry R&D, respectively. Equations A.1 - A.5, B.1 – B.5, and C.1 – C.5 clarify the estimations for each set of instruments (where the instrument is denoted by *w*) corresponding to Equations A – C, respectively. We present the functional relationships for each set in turn. For the first outcome, state and local R&D, we estimate Equations A and A.1 – A.5 as follows:

$\left( A \right) {\Delta State \& Local R\&D}_{int}= \beta_{1}{(\Delta Federal R\&D}_{int})+\beta_{2}{(\Delta State \& Local R\&D}_{int-1})+\beta_{3}\left( \Delta{Nonprofit R\&D}_{int} \right)+\beta_{4}\left( \Delta{Industry R\&D}_{int} \right)+\beta_{5}\left( \Delta{Other R\&D}_{int} \right)+{Year}_{t}+{\Delta\varepsilon}_{1int}$

where,

$$\left( A.1 \right){\Delta Federal R\&D}_{int}= \delta_{1}+\sum_{\mathcal{l=}1}^{4} (\delta_{w1,\mathcal{l}}({Federal R\&D}_{int\mathcal{-l}}))+\varepsilon_{2int}$$

$$\left( A.2 \right) {\Delta State \& Local R\&D}_{int-1}= \delta_{2}+\sum_{k=2}^{4} (\delta_{w2,k}({State \& Local R\&D}_{int-k}))+\varepsilon_{3int}$$

$$\left( A.3 \right) \Delta{Nonprofit R\&D}_{int}= \delta_{3}+\sum_{k=2}^{4} (\delta_{w3,k}(Nonprofit{R\&D}_{int-k}))+\varepsilon_{4int}$$

$$\left( A.4 \right) \Delta{Industry R\&D}_{int}= \delta_{4}+\sum_{k=2}^{4} (\delta_{w4,k}(Industry{R\&D}_{int-k}))+\varepsilon_{5int}$$

$$\left( A.5 \right) \Delta{Other R\&D}_{int}= \delta_{5}+\sum_{k=2}^{4} (\delta_{w5,k}(Other{R\&D}_{int-k}))+\varepsilon_{6int}$$

and where $\mathcal{l}$ ranges from 1 to 4 ($\mathcal{l\geq}1$) and $k$ ranges from 2 to 4 ($k\geq2$), thus each regressor is instrumented with multiple lags. As discussed in the manuscript, endogenous variables are lagged at least two periods as denoted by $k$, while predetermined variables, in this case Federal R&D, are lagged at least one period as denoted by $\mathcal{l}$.

For the second outcome, nonprofit R&D, we estimate Equations B and B.1 – B.5 as follows:

$\left( B \right){\Delta Nonprofit R\&D}_{int}= \beta_{1}{(\Delta Federal R\&D}_{int})+\beta_{2}{(\Delta Nonprofit R\&D}_{int-1})+\beta_{3}(\Delta{State \& Local R\&D}_{int})+\beta_{4}(\Delta{Industry R\&D}_{int})+\beta_{5}(\Delta{Other R\&D}_{int})+{Year}_{t}+{\Delta\varepsilon}_{1int}$

where,

$$\left( B.1 \right){\Delta Federal R\&D}_{int}= \delta_{1}+\sum_{\mathcal{l=}1}^{4} (\delta_{w1,\mathcal{l}}({Federal R\&D}_{int\mathcal{-l}}))+\varepsilon_{2int}$$

$$\left( B.2 \right) {\Delta Nonprofit R\&D}_{int-1}= \delta_{2}+\sum_{k=2}^{4} (\delta_{w2,k}({Nonprofit R\&D}_{int-k}))+\varepsilon_{3int}$$

$$\left( B.3 \right) \Delta{State \& Local R\&D}_{int}= \delta_{3}+\sum_{k=2}^{4} (\delta_{w3,k}(State \& Local{R\&D}_{int-k}))+\varepsilon_{4int}$$

$$\left( B.4 \right) \Delta{Industry R\&D}_{int}= \delta_{4}+\sum_{k=2}^{4} (\delta_{w4,k}(Industry{R\&D}_{int-k}))+\varepsilon_{5int}$$

$$\left( B.5 \right) \Delta{Other R\&D}_{int}= \delta_{5}+\sum_{k=2}^{4} (\delta_{w5,k}(Other{R\&D}_{int-k}))+\varepsilon_{6int}$$

and where $\mathcal{l}$ ranges from 1 to 4 ($\mathcal{l\geq}1$) and $k$ ranges from 2 to 4 ($k\geq2$).

For the third outcome, industry R&D, we estimate Equations C and C.1 – C.5 as follows:

$\left( C \right){\Delta Industry R\&D}_{int}= \beta_{1}{(\Delta Federal R\&D}_{int})+\beta_{2}{(\Delta Industry R\&D}_{int-1})+\beta_{3}(\Delta{State \& Local R\&D}_{int})+\beta_{4}(\Delta{Nonprofit R\&D}_{int})+\beta_{5}(\Delta{Other R\&D}_{int})+{Year}_{t}+{\Delta\varepsilon}_{1int}$

where,

$$\left( C.1 \right){\Delta Federal R\&D}_{int}= \delta_{1}+\sum_{\mathcal{l=}1}^{4} (\delta_{w1,\mathcal{l}}({Federal R\&D}_{int\mathcal{-l}}))+\varepsilon_{2int}$$

$$\left( C.2 \right) {\Delta Industry R\&D}_{int-1}= \delta_{2}+\sum_{k=2}^{4} (\delta_{w2,k}({Industry R\&D}_{int-k}))+\varepsilon_{3int}$$

$$\left( C.3 \right) \Delta{State \& Local R\&D}_{int}= \delta_{3}+\sum_{k=2}^{4} (\delta_{w3,k}(State \& Local{R\&D}_{int-k}))+\varepsilon_{4int}$$

$$\left( C.4 \right) \Delta{Nonprofit R\&D}_{int}= \delta_{4}+\sum_{k=2}^{4} (\delta_{w4,k}(Nonprofit{R\&D}_{int-k}))+\varepsilon_{5int}$$

$$\left( C.5 \right)\Delta{Other R\&D}_{int}= \delta_{5}+\sum_{k=2}^{4} (\delta_{w5,k}(Other{R\&D}_{int-k}))+\varepsilon_{6int}$$

and where $\mathcal{l}$ ranges from 1 to 4 ($\mathcal{l\geq}1$) and $k$ ranges from 2 to 4 ($k\geq2$).

**Model Specification II: Fixed Effects Models (Detailed Notation of Eq. 2)**

Equations D, E, and F present the detailed notation for the institution-field and year fixed effects model (Eq. 2) with the three respective outcomes: state & local, nonprofits, and industry R&D.

$$\left( D \right){State \& Local R\&D}_{int}=\alpha_{in}+ \beta_{1}({Federal R\&D}_{int})+\beta_{2}({Nonprofit R\&D}_{int})+\beta_{3}({Industry R\&D}_{int})+\beta_{4}({Other R\&D}_{int})+{Year}_{t}+\varepsilon_{int}$$

$$(E){Nonprofit R\&D}_{int}=\alpha_{in}+ \beta_{1}({Federal R\&D}_{int})+\beta_{2}({State \& Local R\&D}_{int})+\beta_{3}({Industry R\&D}_{int})+\beta_{4}({Other R\&D}_{int})+{Year}_{t}+\varepsilon_{int}$$

$$(F){Industry R\&D}_{int}=\alpha_{in}+ \beta_{1}({Federal R\&D}_{int})+\beta_{2}({State \& Local R\&D}_{int})+\beta_{3}({Nonprofit R\&D}_{int})+\beta_{4}({Other R\&D}_{int})+{Year}_{t}+\varepsilon_{int}$$

**Model Specification III: Pooled OLS with inclusion of lagged logged dependent variables (Detailed Notation of Eq. 3)**

Equations G, H, and I present the detailed notation for the pooled OLS model with the inclusion of two lagged logged dependent variables: $Y_{int-1}$ and $Y_{int-2}$ (Eq. 3) with the three respective outcomes: state & local, nonprofits, and industry R&D.

$$\left( G \right) \left( {State \& Local R\&D}_{int} \right)= \beta_{0}+ \beta_{1}\left( {Federal R\&D}_{int} \right)+\beta_{2}\left( {State \& Local R\&D}_{int-1} \right)+\beta_{3}\left( {State \& Local R\&D}_{int-2} \right)+\beta_{4}\left( {Nonprofit R\&D}_{int} \right)+\beta_{5}\left( {Industry R\&D}_{int} \right)+\beta_{6}\left( {Other R\&D}_{int} \right)+{Year}_{t}+\varepsilon_{int}$$

$$\left( H \right) \left( {Nonprofit R\&D}_{int} \right)= \beta_{0}+ \beta_{1}\left( {Federal R\&D}_{int} \right)+\beta_{2}\left( {Nonprofit R\&D}_{int-1} \right)+\beta_{3}\left( {Nonprofit R\&D}_{int-2} \right)+\beta_{4}\left( {State \& Local R\&D}_{int} \right)+\beta_{5}\left( {Industry R\&D}_{int} \right)+\beta_{6}\left( {Other R\&D}_{int} \right)+{Year}_{t}+\varepsilon_{int}$$

$$\left( I \right) \left( {Industry R\&D}_{int} \right)= \beta_{0}+ \beta_{1}\left( {Federal R\&D}_{int} \right)+\beta_{2}\left( {Industry R\&D}_{int-1} \right)+\beta_{3}\left( {Industry R\&D}_{int-2} \right)+\beta_{4}\left( {Nonprofit R\&D}_{int} \right)+\beta_{5}\left( {State \& Local R\&D}_{int} \right)+\beta_{6}\left( {Other R\&D}_{int} \right)+{Year}_{t}+\varepsilon_{int}$$

**Model Specification IV: Alternate Dynamic Panel Model (Detailed Notation of Eq. 4)**

Equations J, K, and L present detailed notation for dynamic panel model (Eq. 4) with the adjusted instrument specification for the set of non-federal regressors for the three outcomes state & local, nonprofit, and industry, respectively. Equations J.1 - J.5, K.1 – K.5, and L.1 – L.5 clarify the estimations for each set of instruments (where the instrument is denoted by *w*) for Equations J, K and L, respectively.

We estimate Equations J and J.1 – J.5 as follows:

$(J){\Delta State \& Local R\&D}_{int}= \beta_{1}{(\Delta Federal R\&D}_{int})+\beta_{2}{(\Delta State \& Local R\&D}_{int-1})+\beta_{3}\left( \Delta{Nonprofit R\&D}_{int} \right)+\beta_{4}\left( \Delta{Industry R\&D}_{int} \right)+\beta_{5}\left( \Delta{Other R\&D}_{int} \right)+{Year}_{t}+{\Delta\varepsilon}_{1int}$

where,

$$\left( J.1 \right){\Delta Federal R\&D}_{int}= \delta_{1}+\sum_{\mathcal{l=}1}^{4} (\delta_{w1,\mathcal{l}}({Federal R\&D}_{int\mathcal{-l}}))+\varepsilon_{2int}$$

$$\left( J.2 \right) {\Delta State \& Local R\&D}_{int-1}= \delta_{2}+\sum_{k=2}^{4} (\delta_{w2,k}({State \& Local R\&D}_{int-k}))+\varepsilon_{3int}$$

$$\left( J.3 \right) \Delta{Nonprofit R\&D}_{int}= \delta_{3}+\sum_{\mathcal{l=}1}^{4} (\delta_{w3,\mathcal{l}}(Nonprofit{R\&D}_{int\mathcal{-l}}))+\varepsilon_{4int}$$

$$\left( J.4 \right) \Delta{Industry R\&D}_{int}= \delta_{4}+\sum_{\mathcal{l=}1}^{4} (\delta_{w4,\mathcal{l}}(Industry{R\&D}_{int\mathcal{-l}}))+\varepsilon_{5int}$$

$$\left( J.5 \right) \Delta{Other R\&D}_{int}= \delta_{5}+\sum_{\mathcal{l=}1}^{4} (\delta_{w5,\mathcal{l}}(Other{R\&D}_{int\mathcal{-l}}))+\varepsilon_{6int}$$

and where $\mathcal{l}$ ranges from 1 to 4 ($\mathcal{l\geq}1$) and $k$ ranges from 2 to 4 ($k\geq2$), thus each regressor is instrumented with multiple lags. We estimate Equations K and K.1 – K.5 as follows:

$\left( K \right){\Delta Nonprofit R\&D}_{int}= \beta_{1}{(\Delta Federal R\&D}_{int})+\beta_{2}{(\Delta Nonprofit R\&D}_{int-1})+\beta_{3}(\Delta{State \& Local R\&D}_{int})+\beta_{4}(\Delta{Industry R\&D}_{int})+\beta_{5}(\Delta{Other R\&D}_{int})+{Year}_{t}+{\Delta\varepsilon}_{1int}$

where,

$$\left( K.1 \right){\Delta Federal R\&D}_{int}= \delta_{1}+\sum_{\mathcal{l=}1}^{4} (\delta_{w1,\mathcal{l}}({Federal R\&D}_{int\mathcal{-l}}))+\varepsilon_{2int}$$

$$\left( K.2 \right) {\Delta Nonprofit R\&D}_{int-1}= \delta_{2}+\sum_{k=2}^{4} (\delta_{w2,k}({Nonprofit R\&D}_{int-k}))+\varepsilon_{3int}$$

$$\left( K.3 \right) \Delta{State \& Local R\&D}_{int}= \delta_{3}+\sum_{\mathcal{l=}1}^{4} (\delta_{w3,\mathcal{l}}(State \& Local{R\&D}_{int\mathcal{-l}}))+\varepsilon_{4int}$$

$$\left( K.4 \right) \Delta{Industry R\&D}_{int}= \delta_{4}+\sum_{\mathcal{l=}1}^{4} (\delta_{w4,\mathcal{l}}(Industry{R\&D}_{int\mathcal{-l}}))+\varepsilon_{5int}$$

$$\left( K.5 \right) \Delta{Other R\&D}_{int}= \delta_{5}+\sum_{\mathcal{l=}1}^{4} (\delta_{w5,\mathcal{l}}(Other{R\&D}_{int\mathcal{-l}}))+\varepsilon_{6int}$$

and where $\mathcal{l}$ ranges from 1 to 4 ($\mathcal{l\geq}1$) and $k$ ranges from 2 to 4 ($k\geq2$). We estimate Equations L and L.1 – L.5 as follows:

$\left( L \right){\Delta Industry R\&D}_{int}= \beta_{1}{(\Delta Federal R\&D}_{int})+\beta_{2}{(\Delta Industry R\&D}_{int-1})+\beta_{3}(\Delta{State \& Local R\&D}_{int})+\beta_{4}(\Delta{Nonprofit R\&D}_{int})+\beta_{5}(\Delta{Other R\&D}_{int})+{Year}_{t}+{\Delta\varepsilon}_{1int}$

where,

$$\left( L.1 \right){\Delta Federal R\&D}_{int}= \delta_{0}+\sum_{\mathcal{l=}1}^{4} (\delta_{w1,\mathcal{l}}({Federal R\&D}_{int\mathcal{-l}}))+\varepsilon_{2int}$$

$$\left( L.2 \right) {\Delta Industry R\&D}_{int-1}= \delta_{0}+\sum_{k=2}^{4} (\delta_{w2,k}({Industry R\&D}_{int-k}))+\varepsilon_{3int}$$

$$\left( L.3 \right) \Delta{State \& Local R\&D}_{int}= \delta_{0}+\sum_{\mathcal{l=}1}^{4} (\delta_{w3,\mathcal{l}}(State \& Local{R\&D}_{int\mathcal{-l}}))+\varepsilon_{4int}$$

$$\left( L.4 \right) \Delta{Nonprofit R\&D}_{int}= \delta_{0}+\sum_{\mathcal{l=}1}^{4} (\delta_{w4,\mathcal{l}}(Nonprofit{R\&D}_{int\mathcal{-l}}))+\varepsilon_{5int}$$

$$\left( L.5 \right) \Delta{Other R\&D}_{int}= \delta_{0}+\sum_{\mathcal{l=}1}^{4} (\delta_{w5,\mathcal{l}}(Other{R\&D}_{int\mathcal{-l}}))+\varepsilon_{6int}$$

and where $\mathcal{l}$ ranges from 1 to 4 ($\mathcal{l\geq}1$) and $k$ ranges from 2 to 4 ($k\geq2$).
