# Supplementary material for: The Domino Effects of Federal Research Funding
Source: PLoS One. 2016 Jun 21;11(6):e0157325. doi: 10.1371/journal.pone.0157325 (PMC4915724; doi:10.1371/journal.pone.0157325)
Supplement: S1 Table — (DOCX) [file pone.0157325.s004.docx]

# S1 Table. Descriptive Statistics for Elasticity Computation.

|  | **Full Sample** | **Eng.** | **Physical**  **Science** | **Env.**  **Science** | **Math & Comp.** | **Life**  **Science** | **Social & Psych.** |
| --- | --- | --- | --- | --- | --- | --- | --- |
| Total | 14030.45 | 9646.869 | 7778.866 | 7436.879 | 5633.407 | 45519.14 | 3999.291 |
| Federal | 8834.567 | 6055.437 | 5682.134 | 5019.907 | 4153.706 | 27857.49 | 2165.227 |
| State & Local | 734.8448 | 554.7598 | 146.3701 | 375.2521 | 152.8069 | 2569.548 | 259.7503 |
| Industry | 729.848 | 777.287 | 203.62 | 336.2722 | 214.2158 | 2330.427 | 90.97945 |
| Nonprofit | 778.7174 | 297.6703 | 269.8618 | 245.6203 | 116.533 | 3085.512 | 353.7539 |
| University | 2683.441 | 1765.778 | 1369.991 | 1312.709 | 911.1085 | 8764.56 | 1060.346 |
| Other | 269.029 | 195.9371 | 106.8887 | 147.1188 | 85.03734 | 911.6028 | 69.23442 |
| *Observations* | *17300* | *4605* | *2585* | *1720* | *1920* | *3100* | *3370* |
| ***Public Universities*** | | | | | | | |
| Total | 12906.78 | 9209.867 | 7316.714 | 7916.228 | 5186.696 | 39452.95 | 4157.901 |
| Federal | 7571.263 | 5207.324 | 5019.445 | 5241.011 | 3555.116 | 22433.88 | 2223.058 |
| State & Local | 891.1185 | 691.2519 | 189.6827 | 445.7998 | 208.0371 | 2999.604 | 324.4477 |
| Industry | 632.6934 | 812.4808 | 198.7737 | 329.4653 | 182.1058 | 1729.09 | 90.80998 |
| Nonprofit | 640.8407 | 285.3254 | 261.6634 | 247.1963 | 106.8573 | 2399.025 | 267.7888 |
| University | 2897.953 | 2034.65 | 1534.382 | 1490.321 | 1044.065 | 8962.671 | 1186.429 |
| Other | 272.9098 | 178.8349 | 112.7677 | 162.4335 | 90.51523 | 928.6854 | 65.36706 |
| *Observations* | *12585* | *3355* | *1815* | *1340* | *1345* | *2295* | *2435* |
| ***Private Universities*** | | | | | | | |
| Total | 17029.68 | 10819.78 | 8868.222 | 5746.545 | 6678.323 | 62813.44 | 3586.229 |
| Federal | 12206.5 | 8331.772 | 7244.188 | 4240.223 | 5553.887 | 43319.86 | 2014.619 |
| State & Local | 317.7281 | 188.415 | 44.27612 | 126.4786 | 23.61621 | 1343.487 | 91.26044 |
| Industry | 989.1673 | 682.8271 | 215.0435 | 360.2752 | 289.3253 | 4044.797 | 91.42078 |
| Nonprofit | 1146.73 | 330.8039 | 289.1868 | 240.0627 | 139.1656 | 5042.638 | 577.631 |
| University | 2110.877 | 1044.125 | 982.4971 | 686.3913 | 600.1058 | 8199.758 | 731.9917 |
| Other | 258.6706 | 241.8394 | 93.03092 | 93.11421 | 72.22383 | 862.9013 | 79.30609 |
| *Observations* | *4715* | *1250* | *770* | *380* | *575* | *805* | *935* |
| ***High Capacity Research Units*** | | | | | | | |
| Total | 37630.15 | 23562.32 | 20659.16 | 18827.67 | 15353.05 | 139787.4 | 9646.39 |
| Federal | 24027.3 | 14984.58 | 15408.77 | 12887.76 | 11485.63 | 86757.36 | 5354.394 |
| State & Local | 1839.632 | 1258.093 | 254.3119 | 827.6597 | 402.5003 | 7475.203 | 574.909 |
| Industry | 2037.686 | 1943.995 | 533.2482 | 882.3312 | 637.7301 | 7535.06 | 219.4125 |
| Nonprofit | 2182.299 | 743.9099 | 760.1792 | 650.6338 | 324.879 | 9824.197 | 874.76 |
| University | 6804.732 | 4126.361 | 3395.333 | 3191.491 | 2251.045 | 25376.6 | 2465.498 |
| Other | 738.5005 | 505.3824 | 307.3144 | 387.7971 | 251.2667 | 2818.963 | 157.4168 |
| *Observations* | *5330* | *1485* | *750* | *535* | *595* | *860* | *1105* |
| ***Low Capacity Research Units*** | | | | | | | |
| Total | 3521.974 | 3023.647 | 2514.44 | 2294.199 | 1268.737 | 9326.874 | 1244.305 |
| Federal | 2069.548 | 1805.506 | 1706.669 | 1467.753 | 861.2571 | 5244.156 | 609.3644 |
| State & Local | 242.9054 | 220.0001 | 102.2521 | 171.0006 | 40.68045 | 686.1264 | 105.9974 |
| Industry | 147.4941 | 221.9791 | 68.89462 | 89.73918 | 24.03393 | 332.2195 | 28.32225 |
| Nonprofit | 153.731 | 85.27737 | 69.45965 | 62.76611 | 22.9738 | 498.3378 | 99.57654 |
| University | 848.3127 | 642.2309 | 542.194 | 464.4824 | 309.4012 | 2386.722 | 374.8307 |
| Other | 59.98282 | 48.65305 | 24.97081 | 38.45813 | 10.39094 | 179.3125 | 26.21388 |
| *Observations* | *11970* | *3120* | *1835* | *1185* | *1325* | *2240* | *2265* |

*Notes:* Average Funding, Real (Deflated) Values in $1,000s.

*Key:* Eng. Refers to Engineering, Env. Science refers to Environmental Sciences, Math & Comp. refers to mathematics and computer sciences, and Social & Psych. Refers to Social Sciences and Psychology.
